# Carbon footprint of common procedures in inflammatory bowel disease

**DOI:** 10.1007/s10151-025-03123-5

**Published:** 2025-05-30

**Authors:** L. Munster, B. van der Zwet, J. de Groof, M. Mundt, O. van Ruler, G. D’Haens, W. Bemelman, C. Buskens, M. Duijvestein, T. Stobernack, J. van der Bilt

**Affiliations:** 1 Department of Surgery, Flevoziekenhuis, Almere, The Netherlands; 2 Department of Surgery, Amsterdam UMC (Location VUmc), Amsterdam, The Netherlands; 3 Department of Gastroenterology and Hepatology, Flevoziekenhuis, Almere, The Netherlands; 4 Department of Surgery, IJsselland Hospital, Erasmus University Medical Center, Rotterdam, The Netherlands; 5 Department of Gastroenterology and Hepatology, Amsterdam UMC (Location VUmc), Amsterdam, The Netherlands; 6 Department of Gastroenterology and Hepatology, Radboud University Medical Center, Nijmegen, The Netherlands; 7 Department of Intensive Care Medicine, Radboud University Medical Center, Nijmegen, The Netherlands

**Keywords:** Crohn’s disease, Laparoscopic ileocecal resection, Laparoscopic subtotal colectomy, LIFT, Carbon footprint, Sustainability

## Abstract

**Background:**

The aim of this study was to assess the environmental impact, primarily the carbon footprint of the most common procedures in inflammatory bowel disease (IBD).

**Methods:**

In this study, all processes and products used during a total of eight laparoscopic ileocecal resections (ICRs) in patients with Crohn’s disease (CD), eight laparoscopic subtotal colectomies (STCs) for ulcerative colitis (UC), and eight ligation of the intersphincteric fistula tract (LIFT) procedures in patients with Crohn’s perianal fistula (PAF) (all in adults ≥ 16 years) between March 2023 and May 2024 were collected. A life cycle assessment (LCA) was conducted, mean CO^2^ emission rates were calculated, the major contributors (“hotspots”) were determined, and midpoint/endpoint analysis was performed.

**Results:**

The mean total carbon footprints of laparoscopic ICR, STC, and LIFT were, respectively, 104 kg, 116 kg, and 43.6 kg CO^2^eq, equaling one-way trips by airplane from Amsterdam to Paris, to Manchester, and to Düsseldorf, respectively. The main contributors in laparoscopic ICR and STC were transport of employees and patients (48% and 49%, respectively), energy use in the theater (21% and 27%, respectively), and the use of surgical equipment (14% and 17%, respectively). In LIFT procedures, transport of employees/patients accounted for 47% of total emission rates, followed by the use of surgical equipment (28%), and electricity use in the theater (13%). Besides the impact on global warming, significant impact on fine particulate matter formation, land use, terrestrial acidification, and fossil resource scarcity was identified. Endpoint analysis showed an amount of disability-adjusted life years (DALYs) of approximately 2 h of health damage per laparoscopic ICR/STC and 47 min per LIFT.

**Conclusions:**

The carbon footprint of three commonly performed IBD surgeries is mainly determined by transportation of patients/healthcare personnel, followed by electricity and material use. The latter two vary with the complexity of the surgeries. IBD surgeons should focus on minimizing energy resources and using standard surgical materials. Also, employees should be encouraged to travel by foot/bicycle/public transport/carpooling/electric car.

**Supplementary Information:**

The online version contains supplementary material available at 10.1007/s10151-025-03123-5.

## Introduction

According to the World Health Organization (WHO), climate change, including global warming, is one of the largest healthcare threats of the twenty-first century due to the generation of so-called greenhouse gases (GHG), such as carbon dioxide (CO^2^) [1]. As a paradox, human activities and the healthcare system itself are (partly) responsible for generation of those GHG [2, 3]. Healthcare emissions are estimated to contribute to a combined 5% of global net GHG emissions [2, 4], which is comparable in the UK to an estimated amount of 3–4%, and is even higher in Australia (7%) [5] and the USA (10%) [6]. Although the actual amount of CO^2^ emission in healthcare is still a matter of debate, with varying numbers over time in all different countries, there is a significantly growing interest in sustainable healthcare that minimizes the carbon footprint [7–9].

Within hospitals, surgical theaters are held responsible for the production of a significant amount of direct (e.g., gas, electricity) as well as indirect (e.g., the use of many supplies or production of waste) GHG [4, 10, 11]. Studies on the CO^2^ footprint of several surgical procedures (e.g., laparoscopic right hemicolectomy, cataract surgeries, total knee replacements, or surgery in general) [12–16] have enabled surgeons to reduce (in)direct emission rates by the development of surgery-specific CO^2^-reduction strategies [16]. Moreover, nowadays, in clinical decision-making between different treatment options with similar clinical results, the environmental impact is considered a new but growing outcome parameter alongside quality of life (QoL) and cost-effectiveness to tailor healthcare decisions. This may even be more relevant in the context of chronic disease, such as inflammatory bowel disease (IBD), where the cumulative environmental impact of treatments can be substantial. Recently, the LIR!C trial showed that laparoscopic ileocecal resection (ICR) can be considered an alternative and cost-effective treatment option to infliximab treatment in a subgroup of Crohn’s disease (CD) patients (who presented with limited ileocecal CD and in whom conventional therapy having failed) [17–19]. No data on the GHG production in patients with CD undergoing laparoscopic ICR are to the best of our knowledge available, which is similar to other frequently performed IBD-related surgeries, such as laparoscopic subtotal colectomy (STC) in patients with ulcerative colitis (UC) and the ligation of the intersphincteric fistula tract (LIFT) procedure in patients with Crohn’s perianal fistula (PAF). The aim of this study was to assess the environmental impact, primarily the carbon footprint, of laparoscopic ICR for terminal ileitis in patients with CD, laparoscopic STC in patients with UC, and the LIFT procedure in patients with Crohn’s PAF, and to determine which surgery-specific components contribute the most so as to develop surgery-specific carbon-reduction strategies.

## Materials and methods

### Study design and population

This is a prospective, observational, multicenter cross-sectional study evaluating the CO^2^ generation of laparoscopic ICR for terminal ileitis in patients with CD, laparoscopic STC in patients with UC, and LIFT procedures in patients with Crohn’s PAF. This study was conducted in two nonacademic teaching hospitals (Flevoziekenhuis, Almere, the Netherlands, and IJsselland ziekenhuis, Rotterdam, the Netherlands) between March 2023 and May 2024. On the basis of to previous studies [12–16] and the exploratory nature of this study, eight laparoscopic ICRs, eight laparoscopic STCs, and eight LIFT procedures (*n* = 24 surgeries in total) were considered a minimal sample size in this study to provide a reproducible calculation and general overview of the CO^2^(-equivalent) generation as a result of these surgeries.

This study was conducted according to the Strengthening the Reporting of Observational Studies in Epidemiology (STROBE) guidelines for cross-sectional studies.

### Inclusion and exclusion criteria

All processes and products used during a total of eight laparoscopic ICRs for terminal ileitis, eight laparoscopic STCs in patients with UC, and eight LIFT procedures in patients with Crohn’s PAF (all in adult patients ≥ 16 years) between March 2023 and May 2024 were prospectively observed by two researchers (L.M. and B.Z.) in both participating centers. Exclusion criterium was the conversion of a laparoscopic ICR or laparoscopic STC into an open resection.

### Ethical approval

This study did not fall within the scope of the Medical Research Involving Human Subjects Act (WMO), since patients were only indirectly involved in this study. Therefore, ethical approval by a medical ethics review committee (MERC) was not obtained. However, this project was conducted according to the principles of the Declaration of Helsinki (64th WMA General Assembly, Fortaleza, Brazil, October 2013) and according to the General Data Protection Regulation (GDPR).

### Life cycle assessment (LCA)

In healthcare, life cycle assessments (LCAs) are used as a tool to evaluate and compare the environmental impact, including carbon footprint, of all products and processes. This methodology has experienced a significant growth in the past decade and has been applied to similar processes of healthcare [20–22]. LCAs are internationally standardized approaches enabling the quantification of materials and energy input and output, throughout the lifecycle of a predefined study object. LCAs also allow to assess the environmental impact of regional and global resources to evaluate its depletion and environmental degradation, thereby representing the environmental implications of human activities [23].

 Typically, LCAs involve four phases. In the first phase, the scope (including system boundaries) and eventual goals of the LCA are defined. Second, an inventory analysis for all processes in the life cycle, including all relevant (technical) in-/outputs, emissions, and the use of resources, is conducted. The inventory of the current study was created by using the EcoInvent database (version 3.9) as described at https://ecoinvent.org/the-ecoinvent-database (*Accessed February 21, 2024*). In the third phase of the LCA, the impact of all (grouped) resources and emissions are evaluated and quantified to create comparable groups. In the last phase, results of the environmental impact findings are interpreted [23]. In this way, potential so-called hot spots according the environmental impact can be identified [23, 24]. The SimaPro software package (version 9.5.0.2), including the ReCipe 2016 Endpoint (h) V1.08 / World (2010) H/A method, was used to conduct midpoint as well as endpoint analysis in this LCA [25, 26]. Midpoint analyses are primarily focused on separate environmental details (e.g., water consumption or global warming), whereas endpoint analyses are focused on the general effect and damage on three aggregation levels (also known as areas of protection): the impact on our ecosystem (in extinct species per year), human health (in disability-adjusted life years [DALYs]), and resources (in US dollars) [25, 26], in this study as a result of one surgery. DALYs represent the comprehensive disease burden in a population, indicative of the total number of years lost due to disability, premature death, or poor health conditions [27].

### System boundary and data collection

**Fig. 1:**
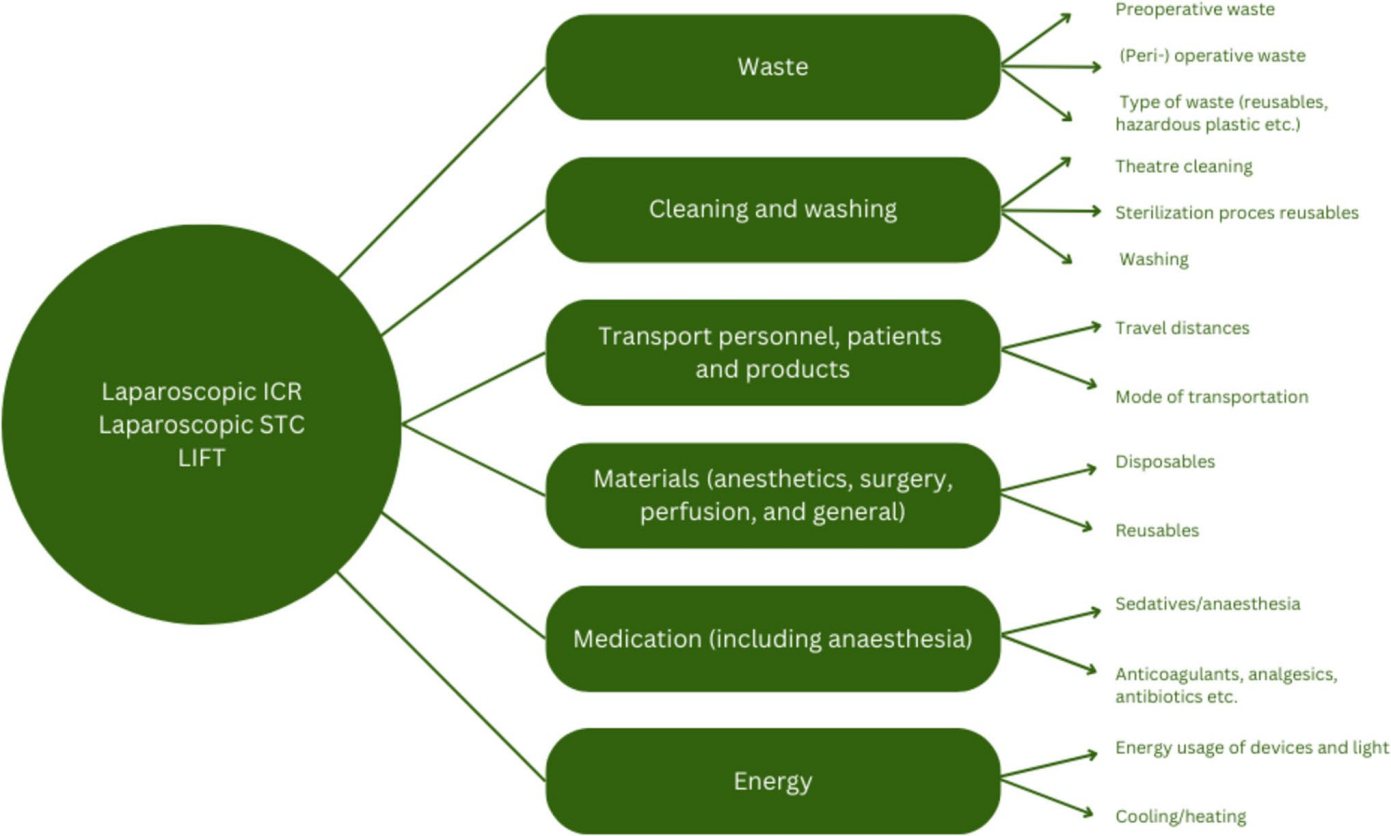
System boundary

As mentioned above, the first phase of the LCA included a predefined system boundary (Fig. 1). This system boundary included all specific (peri-)operative processes of a laparoscopic ICR, a laparoscopic STC, and a LIFT procedure, including materials (e.g., disposables and reusables), energy (e.g., power usage of devices and light, cooling/heating), medication/anesthetics (e.g., sedative medication, analgesics, antibiotics, or anticoagulants), waste (pre-, intra- and postoperative, including the type of waste), and cleaning/washing (e.g., cleaning of the theater, sterilization of reusables, and washing of surgery clothing). The system boundary served as reference in the data collection process. In the second phase, an inventory list based on the system boundary was created, including all relevant data that needed to be observed during all surgeries by two researchers (L.M. and B.Z.), and checked by L.M. This input list covered both fixed and variable elements, and all stages in the LCA (production and distribution of all products) were considered. For all items used during a laparoscopic ICR, a laparoscopic STC, and a LIFT procedure, the following data were recorded: materials, weight/volume, manufacturing place, supplier location, distributor location, distance from distributor to the hospital, transportation means and prices. Modes of transportation were recorded, and traveling distances from and to the hospital of both staff and patients were calculated by the use of Google Maps (https://www.google.nl/maps). Afterward, those modes and distances were converted into CO^2^ equivalents (kg) by the use of SimaPro and the ReCipe method as mentioned above. As employees are expected to be at work regardless of whether a laparoscopic ICR, STC, or LIFT is scheduled, and mobility emission rates could vary within hospitals depending on urban or rural localization and hence accessibility, we separately analyzed the environmental impact of the procedure itself, i.e., without transport of personnel and patient, to improve comparability (e.g., to compare the environmental impact of treatments with similar clinical results).

Waste consumption data were derived from proxy data from Radboud University Medical Center (Radboudumc), the Netherlands, as well as missing data (or reported if not possible). Energy consumption in the theater was measured using a standard in hospital energy gauge (kilowatt per hour). Food and drinks as consumed by the patients and staff were not considered. Similarly, details on construction of the hospital and the production of machines were excluded.

To facilitate comparable and straightforward CO^2^ equivalent emission rates, a comparison was made between the outcomes in this study and traveling distances by airplane or car, using conversion factors provided by https://www.co2emissiefactoren.nl/ (Well-to-Wheel conversion factor of 0.234 kg per kilometer for short-distance flights, and 0.193 kg per kilometer for distances by car).

## Results

**Fig. 2:**
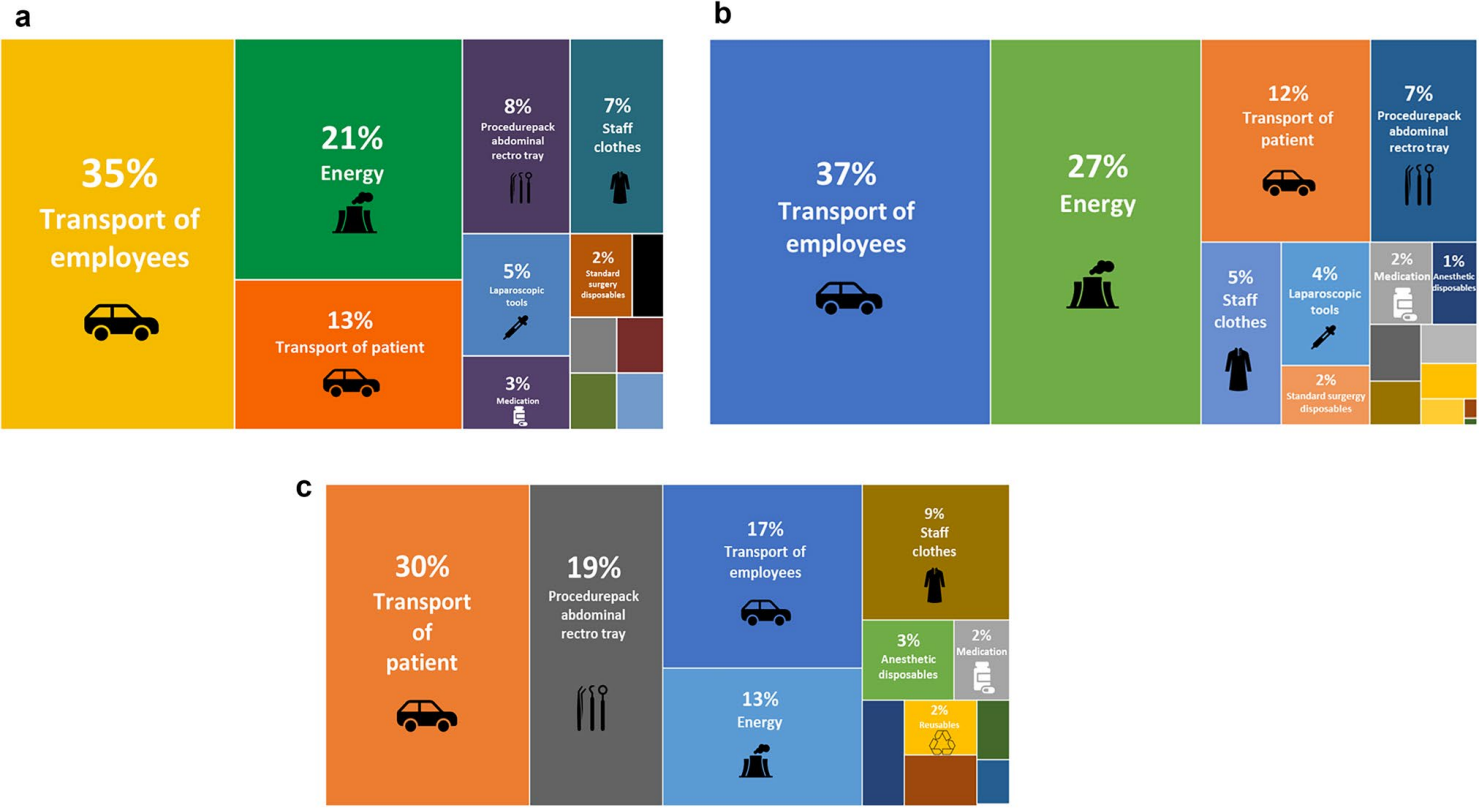
**A** Tree map of the LCA of laparoscopic ICR for terminal ileitis in patients with CD. **B** Tree map of the LCA of laparoscopic STC in UC. **C** Tree map of the LCA of LIFT procedures in patients with Crohn’s PAF

All processes and products involved in a total of eight laparoscopic ICRs for terminal ileitis in patients with CD, eight laparoscopic STCs in patients with UC, and eight LIFT procedures in patients with Crohn’s PAF were analyzed. The mean total carbon footprint was 104 kg CO^2^eq in laparoscopic ICR for terminal ileitis for CD, 116 kg CO^2^eq in laparoscopic STC for UC, and 43.6 kg CO^2^eq in a LIFT procedure in patients with Crohn’s PAF (including transport of staff and patients), which equals one-way trips by airplane from Amsterdam (the Netherlands) to Paris (France; 430 km by air), to Manchester (England; 494 km by air), and to Düsseldorf (Germany; 182 km by air), respectively. Figure 2 presents tree maps of the LCA of ICR for terminal ileitis in patients with CD, laparoscopic STC in patients with UC, and LIFT procedures in patients with Crohn’s PAF, indicating all so-called hotspots in the carbon footprint of one single surgery (including transport of personnel and patients). Supplementary Fig. 1 presents a complete overview of the entire LCA in process trees (including transport of personnel and patients; 1.5% cut-off). The environmental impact analysis showed several main contributors. In laparoscopic ICR these were transport of employees (35%), transport of patients (13%), electricity in the theater (21%), medication (3%), laparoscopic tools (5%), and a combination of standard surgery items (including surgical drapes/equipment covers, staff clothes and standard surgery disposables; 17% combined). The LCA of transport of employees and patients accounted for the largest share of the total carbon footprint (50.2 kg CO^2^eq; 48% combined), followed by energy use in the theater (21.6 kg CO^2^eq; 21%).

In laparoscopic STC main contributors were quite similar as compared with laparoscopic ICR, including transport of employees (37%), transport of patients (12%), electricity in the theater (27%), laparoscopic tools (4%), a combination of standard surgery items (including surgical drapes/equipment covers, staff clothes, and standard surgery disposables; 14% combined), medication (2%), and anesthetic disposables (1%). In this type of surgery, the LCA of transport of employees and patients also accounted for the largest share of the total carbon footprint, and total CO^2^eq emission rates were slightly higher as compared with laparoscopic ICR (56.1 kg CO^2^eq; 49% combined), followed by energy use in the theater (31.9 kg CO^2^eq; 27%. The difference between laparoscopic ICR and STC can mainly be explained by the difference in the duration of the surgery, and consequently the use of theater in general, which was longer in STC.

In LIFT procedures for Crohn’s PAF, transport of patients was a larger contributor (30%) as compared with transport of employees (17%), which was the other way around in laparoscopic ICR and STC. Other main contributors were a combination of standard surgery items (including surgical drapes/equipment covers, staff clothes, and standard surgery disposables; 28% combined), electricity use in the theater (13%), anesthetic disposables (3%), medication (2%), and reusables (2%).

The mean total carbon footprint of a laparoscopic ICR for terminal ileitis without transport of personnel and patients was 54.0 kg CO^2^eq, equaling a one-way trip by car from Amsterdam (the Netherlands) to Cologne (Germany; 264 km by car). In laparoscopic STC this was 60.2 kg CO^2^eq, equaling a one-way trip by car from Amsterdam to Luxembourg (Luxembourg; 361 km by car), and in LIFT procedures this was 23.3 kg CO^2^eq, equaling a trip from Amsterdam to Eindhoven (the Netherlands; 125 km by car). When excluding transport of employees and patients, it was shown that main carbon footprint contributors were electricity use in the theater, including air treatment (heating, ventilation, and air conditioning) and equipment, with a mean estimated 21.6 kg, 31.9 kg, and 5.5 kg CO^2^eq emissions in laparoscopic ICR, STC, and LIFT procedures, respectively, which accounted for 40%, 53%, and 24% of total CO^2^eq emissions, respectively. These emission rates were directly correlated with the duration and complexity of surgery, and consequently the use of theater in general since long and complex procedures require more theater time and material.

In LIFT procedures, the use of surgical equipment accounted for an even larger share, with a combined 12.5 kg CO^2^eq emission rate, which accounted for 54% of the total CO^2^eq emission rates of a LIFT procedure solely (without transport of employees and patients). LCAs, illustrating the impact assessment of all three procedures without transport of personnel and patients, are presented in Supplementary Fig. 2.

### Midpoint analysis

**Fig. 3:**
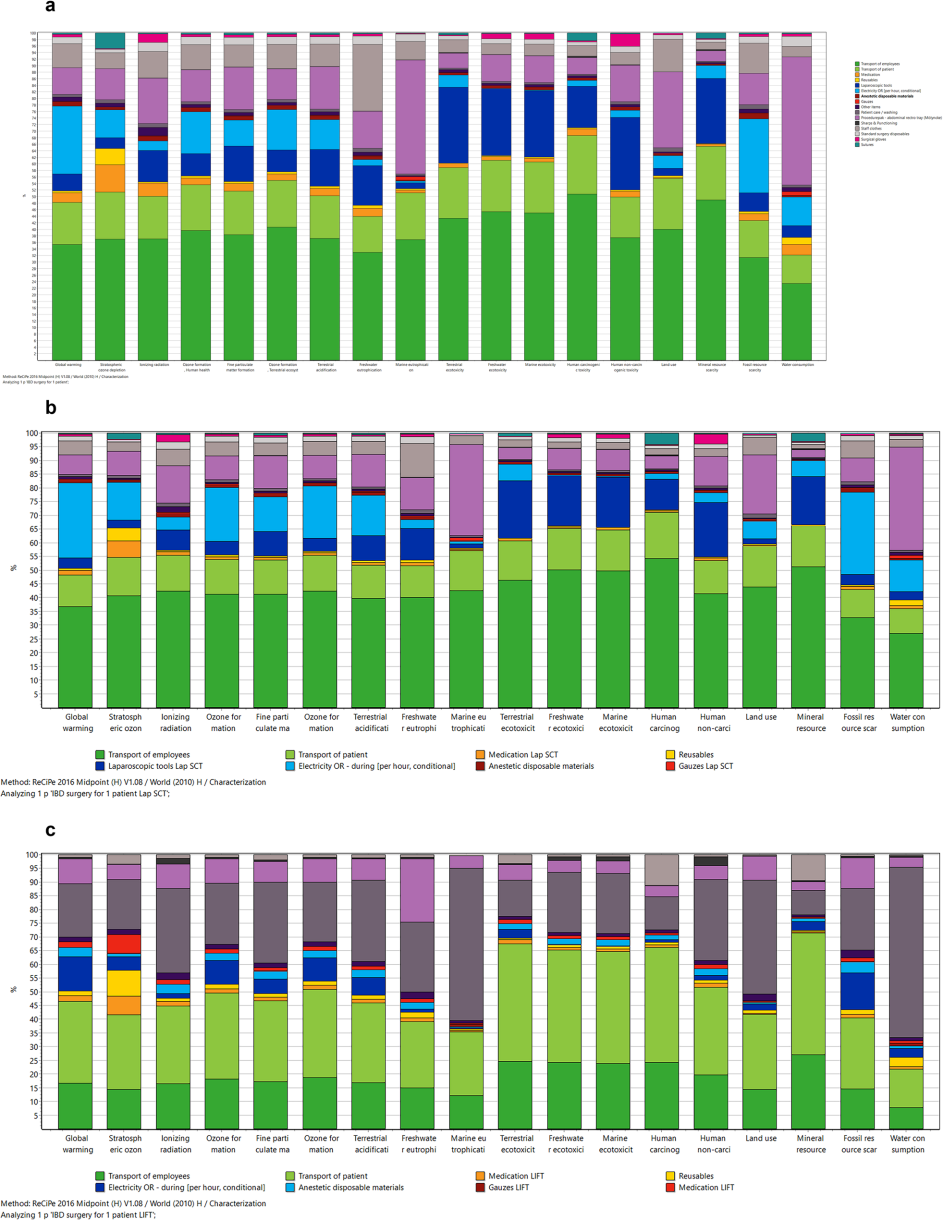
**A** Midpoint analysis of laparoscopic ICR for terminal ileitis in patients with CD. **B** Midpoint analysis of laparoscopic STC in patients with UC. **C** Midpoint analysis of LIFT procedures in patients with Crohn’s PAF

Figure 3 illustrates the midpoint analyses showing the impact of laparoscopic ICR for terminal ileitis in CD, laparoscopic STC in UC, and LIFT procedures in patients with Crohn’s PAF on several specific environmental factors (whereas endpoint analyses provide a more general overview). As mentioned above, transport of employees and patients, as well as energy use in the theater, and the use of surgical equipment are major contributors to CO^2^eq emissions in all three surgeries. Besides the impact of those factors on global warming (86%, 90%, and 88%, respectively, of the total impact on global warming), a significant impact on fine particulate matter formation (82%, 85%, and 89%, respectively, of the total impact on fine particulate matter formation), land use (in all three surgeries 94% of the total impact on land use), terrestrial acidification (81%, 85%, and 90%, respectively, of the total terrestrial acidification), and fossil resource scarcity (86%, 90%, and 87%, respectively, of the total fossil resource scarcity) was identified.

### Endpoint analysis

Endpoint analyses of all three surgeries are shown in Table 1. The amount of DALYs (0.000215 and 0.000235 for laparoscopic ICR and STC, respectively) are equal to approximately 2 h of health damage in one person worldwide per single laparoscopic ICR or laparoscopic STC. For a singe LIFT procedure (with DALY 0.000090), this equals approximately 47 min in one person worldwide. An overview of the damage assessment per category is presented in Supplementary Fig. 3.

**Table 1:**
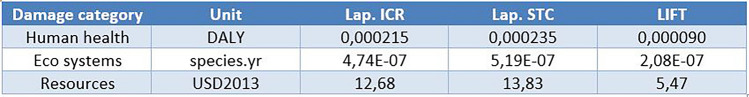
Endpoint analysis

## Discussion

This study showed a mean total carbon footprint of 104 kg CO^2^eq, 116 kg CO^2^eq, and 43.6 kg CO^2^eq in laparoscopic ICR for CD, laparoscopic STC for UC, and LIFT procedures in patients with Crohn’s PAF, respectively. This equals one-way trips by airplane from Amsterdam to Paris, to Manchester, and to Düsseldorf, respectively. Remarkably, the main contributors in laparoscopic ICR and STC were transport of healthcare personnel and patients (48% and 49%, respectively), and energy use in the theater (21% and 27%, respectively). In LIFT procedures, the transport of healthcare personnel and patients accounted for 47% of total emission rates, electricity use in the theater for 13%, and also the use of surgical equipment accounted for a large share (28%). This can be explained by the fact that transportation of patients and medical personal is relatively constant, while electricity and material use depend on the type of procedure. Long and complex procedures require more theater time and material.

The healthcare system, in particular surgical care, is being held responsible for a fair amount of generation of GHG, including CO^2^. Surgical theaters expend three to six times more energy than other departments in hospitals [28, 29], and surgical waste is an important topic of interest in the field of GHG emissions [30]. It is therefore inevitable that surgical associations, for example, the American College of Surgeons, have started to raise more awareness on this topic [31]. Introducing the carbon footprint as a new decisive outcome parameter incentivizes the adoption of more sustainable healthcare practices. It encourages the development and utilization of (medical) technologies, procedures, and treatments that minimize environmental harm without compromising patient care (e.g., minimizing the use of redundant (sedative) medication/items in the theater, the use of renewable energy sources, or telemonitoring, if possible, to reduce transport movements) [31].

To our knowledge this is the first study that assessed the detailed environmental impact of frequently performed IBD-related surgeries such as laparoscopic ICR, laparoscopic STC, and LIFT. Although we do acknowledge that it is challenging to compare these surgeries with each other, the carbon emission rates as a result of laparoscopic ICRs, STCs, and LIFT procedures were calculated using an exhaustive LCA, which could serve as a benchmark for future studies and surgery-specific reduction strategies. Comparing other surgical procedures, as indicated in a systematic review of Rizan et al., showing that the carbon footprint of a single surgery ranged from 6 to 814 kg CO^2^eq, the current study showed that the carbon footprint of a laparoscopic ICR, STC, and LIFT were at the lower end of the spectrum [12]. Taylor et al. showed even lower emission rates in laparoscopic right hemicolectomy (22.21 kg CO^2^eq), which could be explained by the fact that they excluded anesthetics and perioperative materials [16]. Transanal mesorectal excision (TME) for cancer (408,6 kg CO^2^eq) and robotic hysterectomy (814 kg CO^2^eq) are examples at the upper end [12–16, 32, 33]. However, it should be kept in mind that comparing (surgical) GHG emission studies directly is difficult due to heterogeneity and methodological differences between all studies (e.g., various system boundaries) [12, 16]. Also, it should be kept in mind that healthcare protocols potentially differ per hospital or region. As an example, in the current study, it was remarkable that the included hospitals only used total intravenous anesthesia (TIVA) as part of their so-called Green Team initiatives as compared with other (mostly nonacademic) hospitals/studies that often use anesthetic gases during surgery. The latter is inherent to higher healthcare emission rates.

While most studies reported on the carbon footprint (alias global warming) as a result of a specific surgery only, we also provide detailed insight into several other important aspects of the environmental impact, including the impact on fine particulate matter formation, land use, terrestrial acidification, and fossil resource scarcity. Moreover, endpoint analyses showed that laparoscopic ICR and STC resulted in approximately 2 h of health damage per laparoscopic ICR and STC (expressed as DALYs), and 47 min in LIFT procedures, which is indicative for the total number of hours lost due to disability, premature death, or poor health conditions in one person worldwide [27]—paradoxically in this case affecting mainly people from lower-income countries (since the impact of climate change on health is assumed to be much higher in low- versus high-income countries) from where most resources were derived [7–9].

Although awareness is increasing and a variety of initiatives, e.g., so-called Green Teams, have emerge to reduce emission rates in the theaters according to the “5 R principle” (Reduce, Reuse, Recycle, Rethink, and Research; e.g., energy savings, the use of less instruments and textiles, telehealth visits, the use of TIVA instead of anesthetic gases) [13, 34], there is still considerable room for improvement. On the basis of this study, surgeons (and anesthesiologists) should focus on minimizing energy resources and the use of standard surgical products (including surgical drapes/equipment covers, staff clothes, and standard surgery disposables), without compromising safety and outcomes. Especially in less invasive surgeries such as a LIFT procedure (with proportionally shorter time in the theater), it should be kept in mind that the use of surgical equipment accounts for the largest share of CO^2^eq emission rates, and that the use of equipment should be reduced if possible. In line with this, all attending healthcare employees should encourage the use of sustainable alternatives if possible (e.g., reusable surgical caps instead of disposables, although controversially it should be kept in mind that sterilization processes also account for their share in total CO^2^eq emission rates). To reduce electricity use in the theater, the attending surgeon and anesthesiologist should always strive for the shortest time as possible in the theater. We do feel that the importance of the healthcare footprint reduction strategies should be addressed with all surgeons and should be applied during training of young, new surgeons. Last, but definitely not least, this study should empower healthcare professionals and decision-makers to reduce the healthcare footprint simply by encouraging employees (and if possible patients) to travel by foot, bicycle, public transport, carpooling, or electric car.

This study has several limitations inherent to its design. We did not take into account the use of hospital beds, material, and energy expenditure at the ward, which are important for the procedures where there is necessarily an admission. Therefore, to improve comparability with other (pharmaceutical) treatment options (of which data on GHG emission are currently often lacking), it would be recommended to also account for, among others, length of hospital stay and all other equipment used during hospital stay, complications, reinterventions, or admission to the intensive care unit (ICU), including transport of not only all medical stakeholders but also of all visitors. More detailed data on the environmental impact of different treatment modalities in IBD, but also in chronic disease in general, may contribute to sustainable clinical decision-making in the future, particularly if surgery proved to be an alternative to medical management. Lastly, it should be kept in mind that the current study was conducted in rural hospital settings, which may have led to an overrepresentation of travel emissions of patients as well as employees as compared with urban hospital settings.

In conclusion, the carbon footprint of three commonly performed surgeries in IBD is mainly determined by transportation of patients and healthcare personnel, followed by electricity and material use. The latter two vary with the complexity of the surgeries. IBD surgeons should focus on minimizing energy resources and using standard surgical materials. Also, employees should be encouraged to travel by foot, bicycle, public transport, carpooling, or electric car.

## Supplementary Information

Below is the link to the electronic supplementary material.

Supplementary file1 (1967 KB)

Supplementary file2 (70 KB)

## Data Availability

All authors of this manuscript confirm that the data supporting the findings of this study are available within the manuscript. Supplementary details (including a complete overview of the inventory list) are available upon reasonable request.
