# Supplementary material for: Carbon footprint of common procedures in inflammatory bowel disease
Source: Tech Coloproctol. 2025 May 30;29(1):127. doi: 10.1007/s10151-025-03123-5 (PMC12125120; doi:10.1007/s10151-025-03123-5)
Supplement: Supplementary file 1 — Supplementary file1 (1967 KB) [file 10151_2025_3123_MOESM1_ESM.docx]

**Tables**

| Damage category | Unit | Lap. ICR | Lap. STC | LIFT |
| --- | --- | --- | --- | --- |
| Human health | DALY | 0,000215 | 0,000235 | 0,000090 |
| Eco systems | species.yr | 4,74E-07 | 5,19E-07 | 2,08E-07 |
| Resources | USD2013 | 12,68 | 13,83 | 5,47 |

**Table 1** Endpoint analysis focusing on the effect and damage on three

aggregation levels (‘areas of protection’): the impact on our ecosystem (in species per year), human

health (in DALY’s: disability-adjusted life years) and resources (in dollars) as a result of one single

surgery.

**Figures**


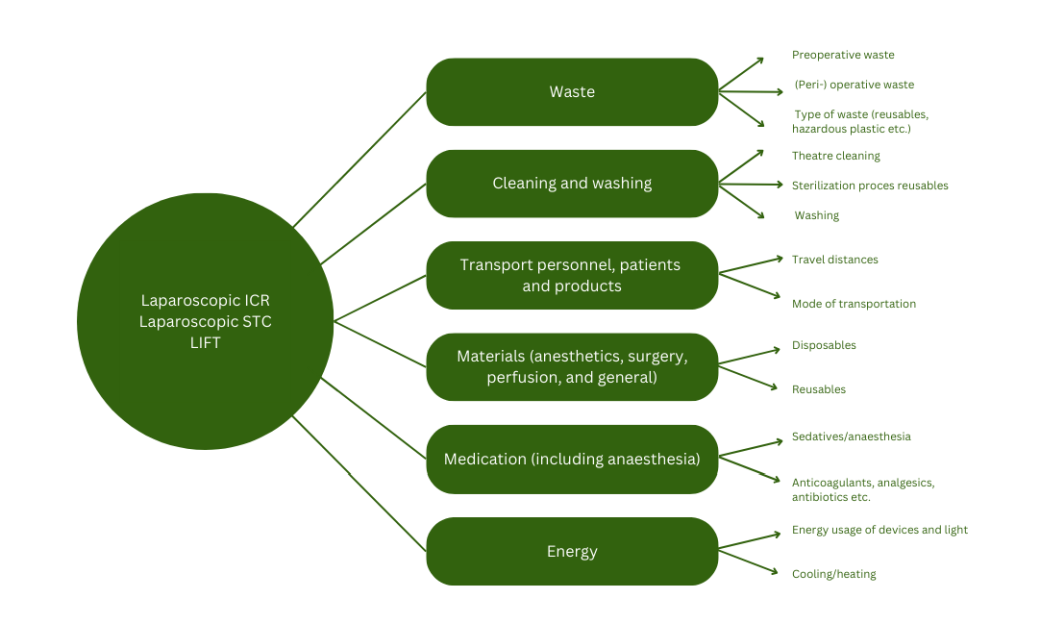


**Figure 1** System boundary showing all included aspects as observed during a single

laparoscopic ICR, a single laparoscopic STC, and a single LIFT procedure

**
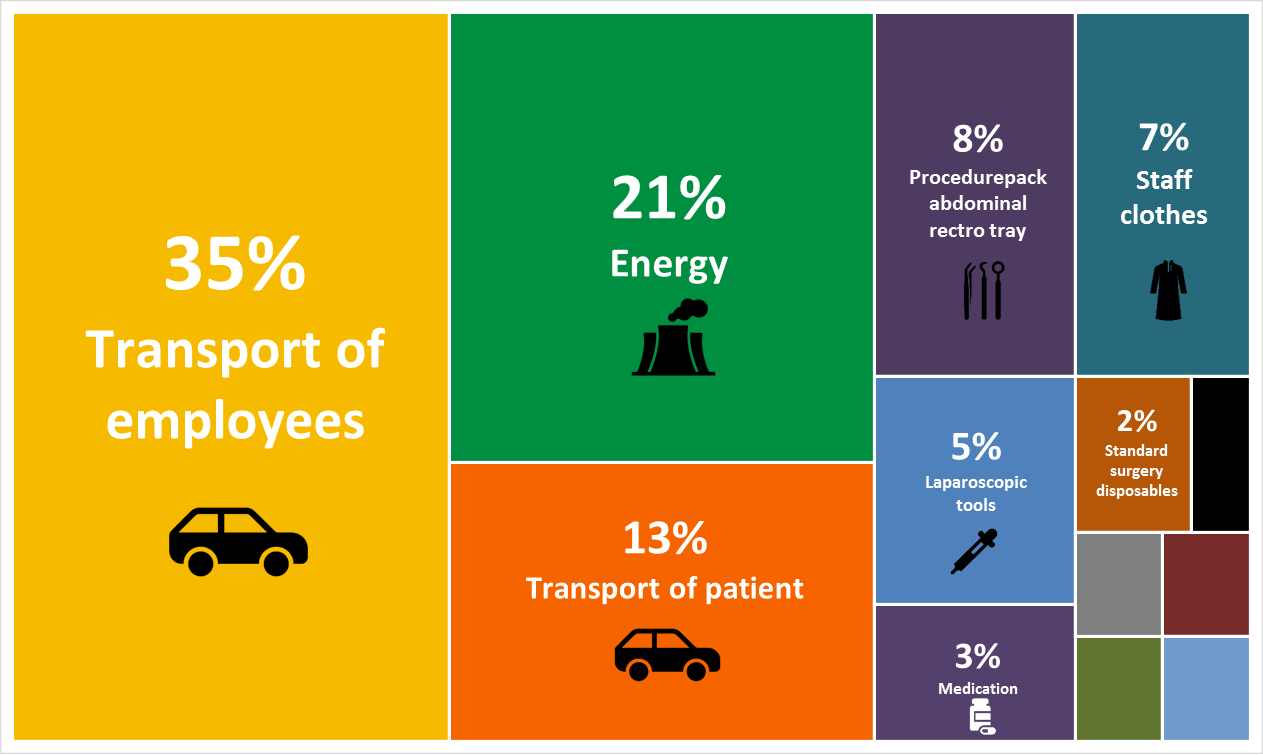
**

**Figure 2a** Tree map of the LCA of laparoscopic ICR for terminal ileitis in CD (total CO2eq emission = 104kg)


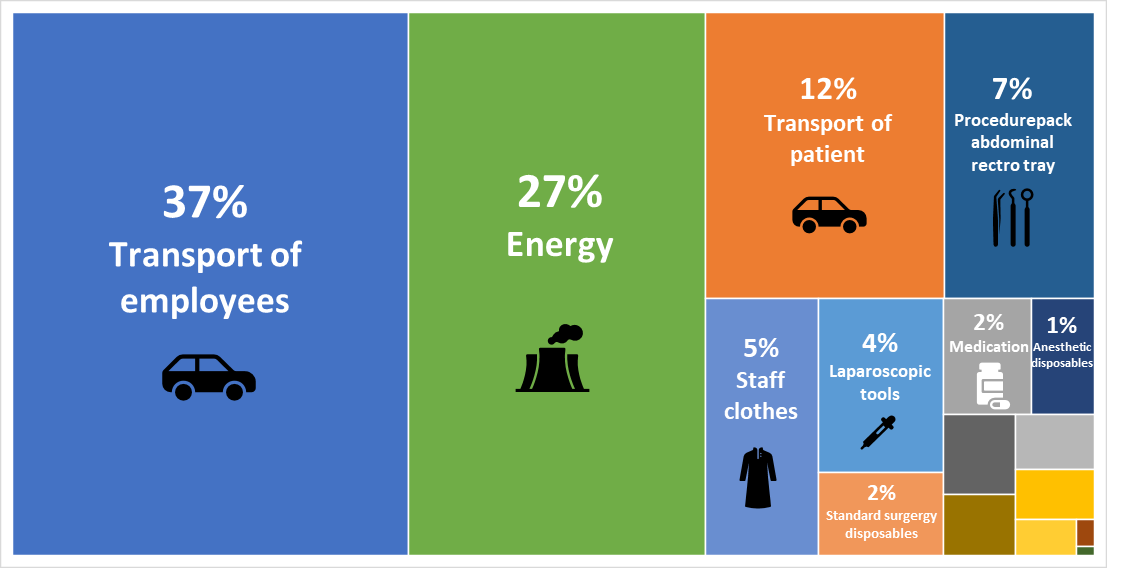


**Figure 2b** Tree map of the LCA of laparoscopic STC in UC patients (total CO2eq emission = 116kg)


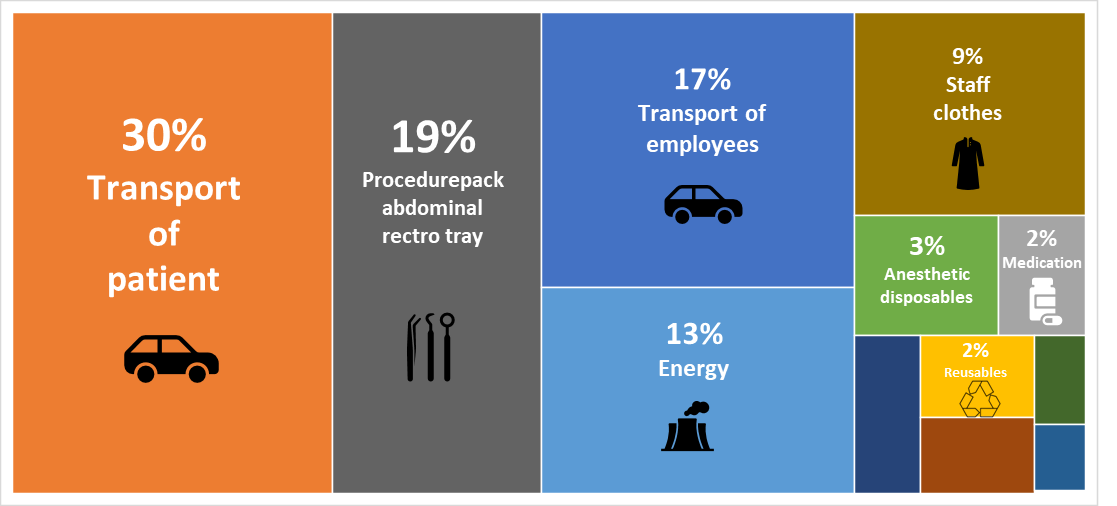


**Figure 2c** Tree map of the LCA of LIFT procedures in Crohn’s PAF patients (total CO2eq emission = 43.6kg)

**
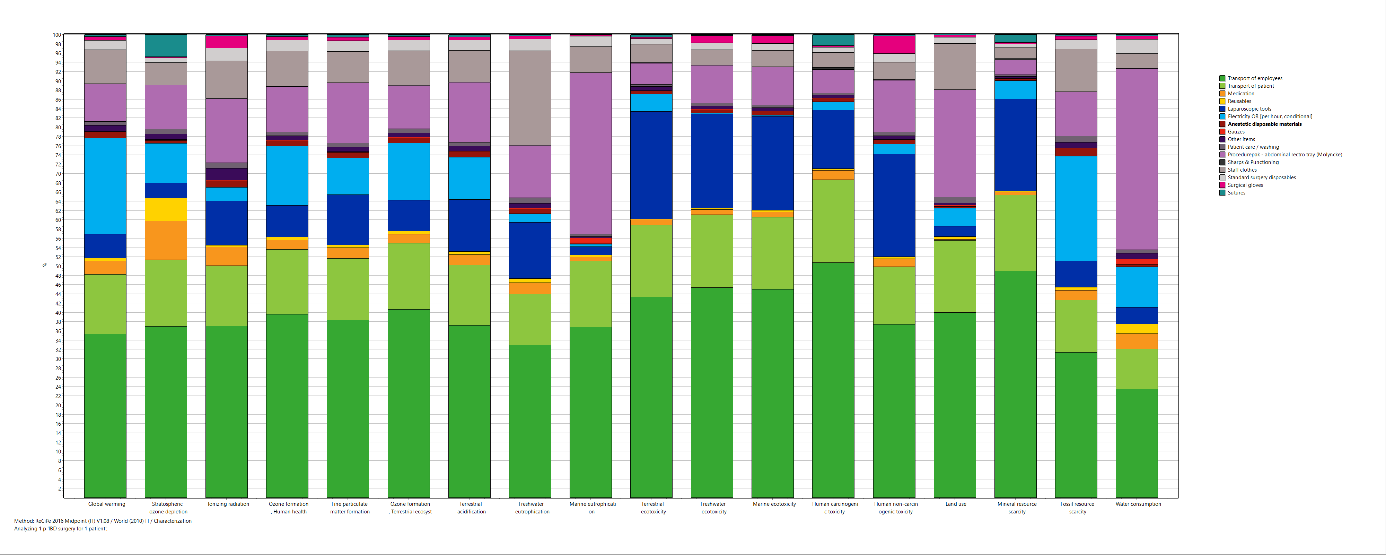
**

**Figure 3a** Midpoint analysis showing the impact on specific environmental factors (e.g. water consumption or global warming) of all separate items from the LCA of one laparoscopic ICR for terminal ileitis in a CD patient

**
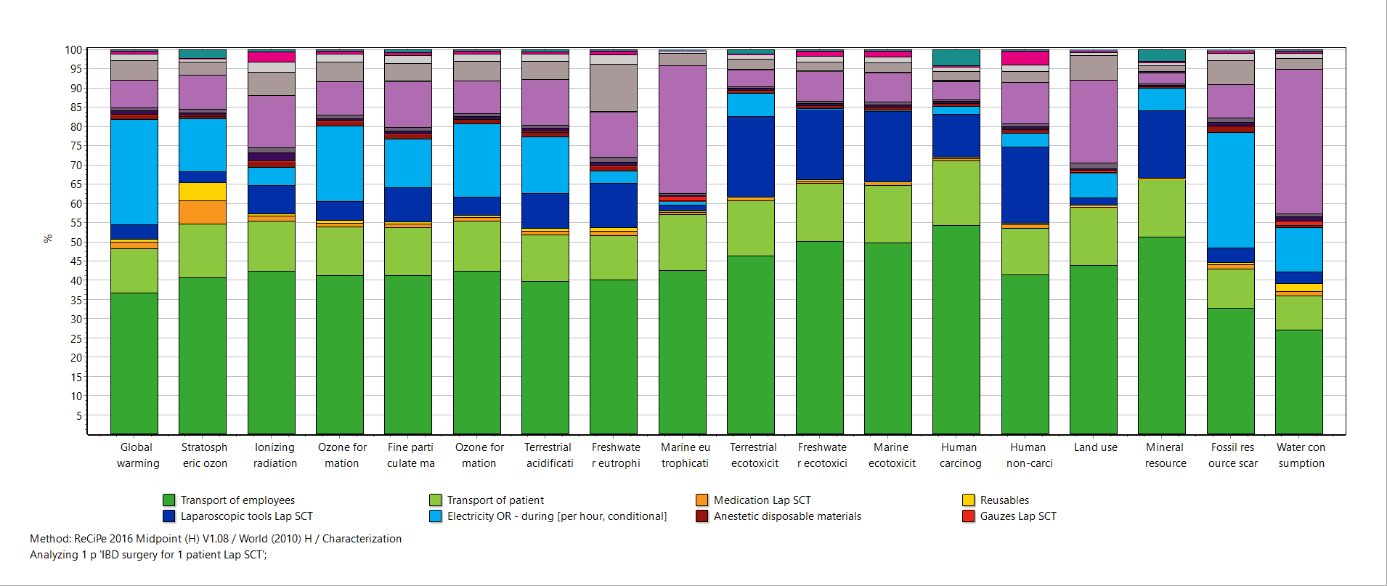
**

**Figure 3b** Midpoint analysis showing the impact on specific environmental factors (e.g. water consumption or global warming) of all separate items from the LCA of one laparoscopic STC in a UC patient


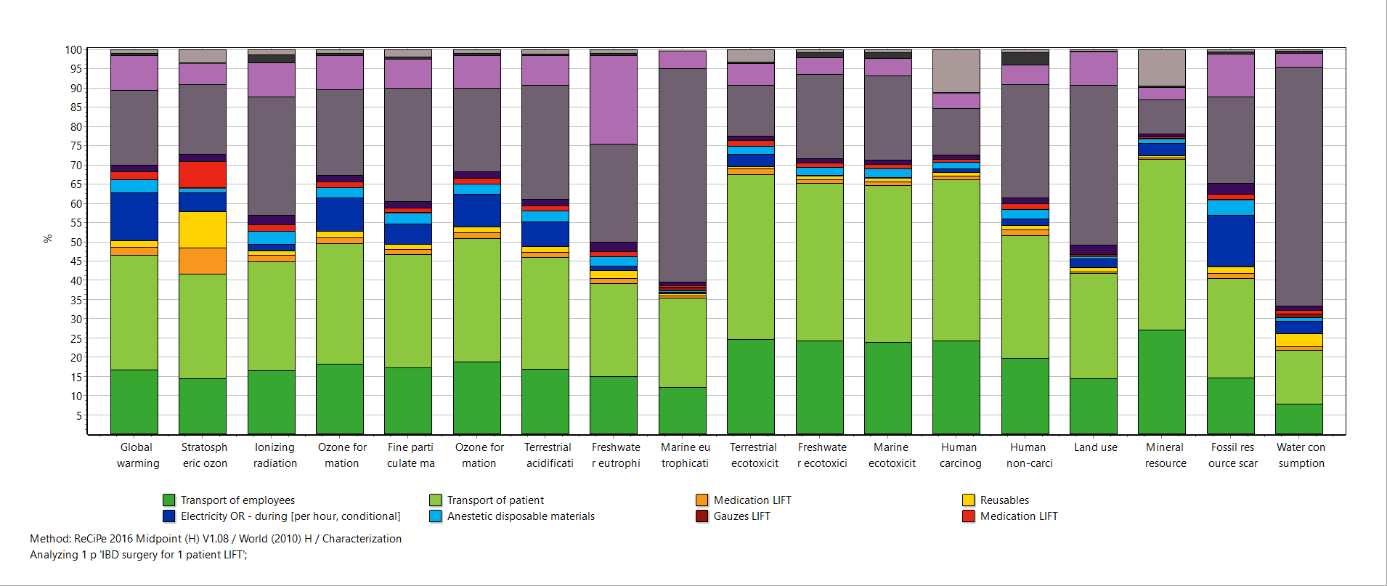


**Figure 3c** Midpoint analysis showing the impact on specific environmental factors (e.g. water consumption or global warming) of all separate items from the LCA of one LIFT procedure in a Crohn’s PAF patient

**Supplementary Figures**

**
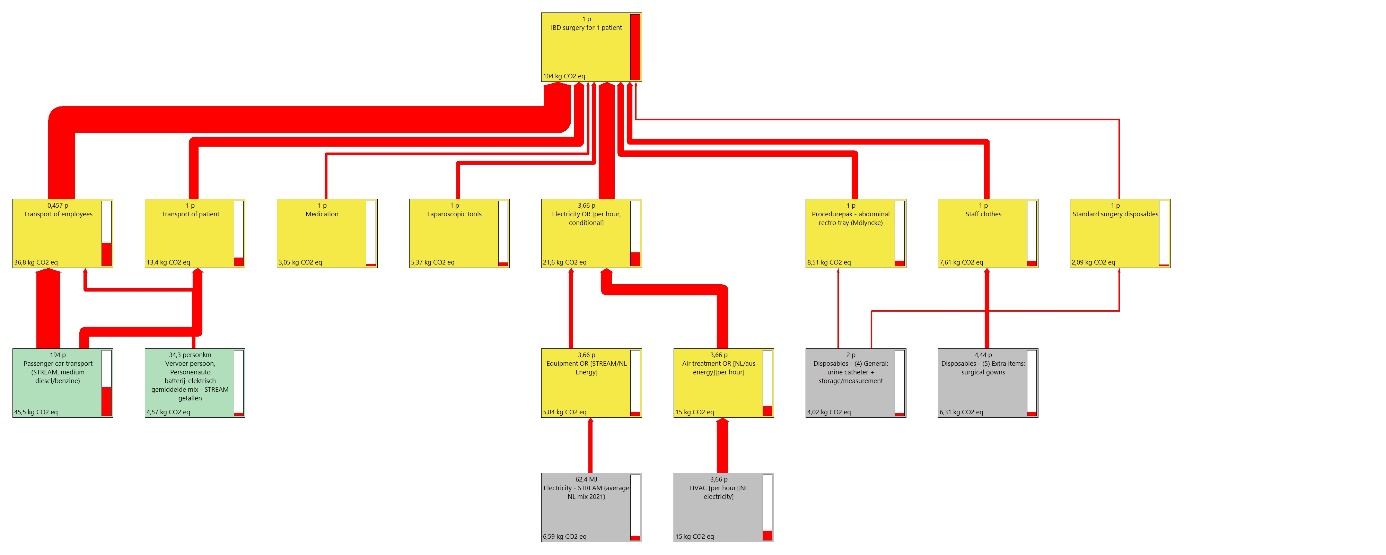
**

**Supplementary Figure 1a** LCA process tree illustrating the impact assessment (1.5% cut-off) expressed in CO2eq of one laparoscopic ICR for terminal ileitis in CD. Thickness of the red lines shows the impact of each input. *Eq: equivalents, GLO: Global Emission Averages, MJ: mega joule, RoW: Rest of World emission averages, HVAC: heating, ventilation and air conditioning.*

**
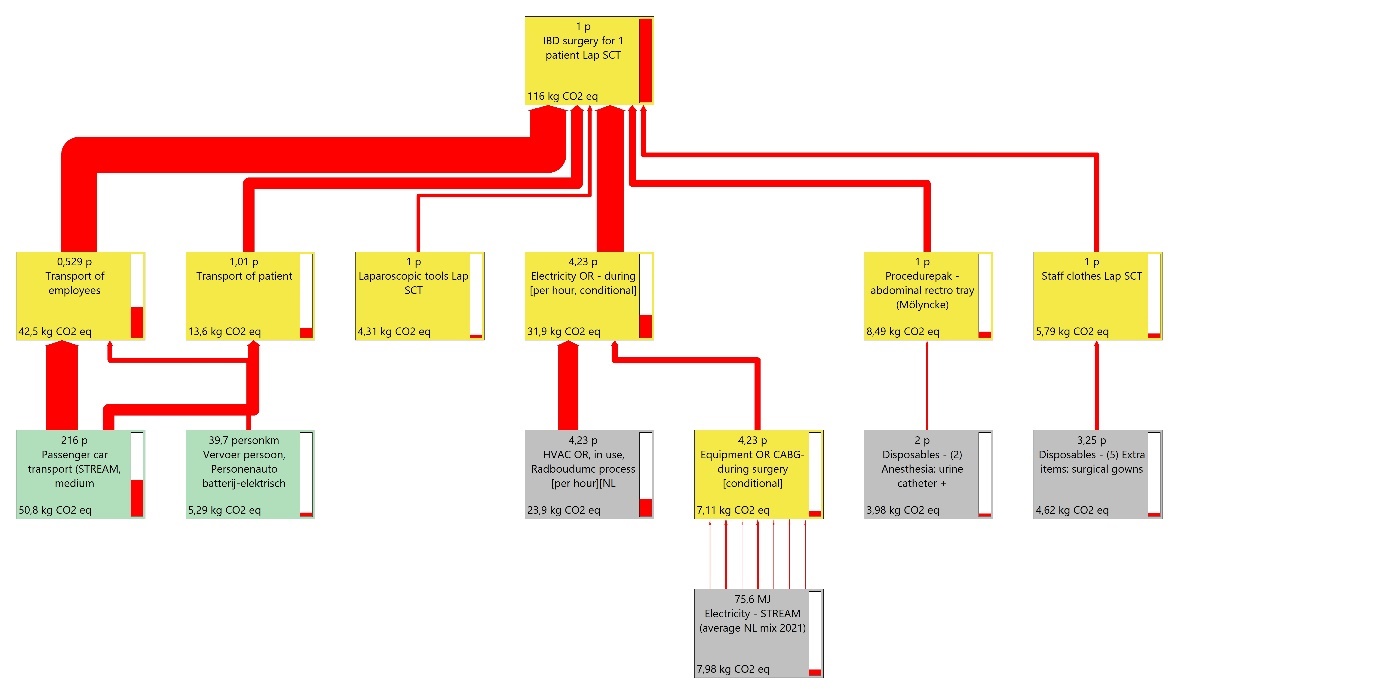
**

**Supplementary Figure 1b** LCA process tree illustrating the impact assessment (1.5% cut-off) expressed in CO2eq of one laparoscopic STC in UC. Thickness of the red lines shows the impact of each input. *Eq: equivalents, GLO: Global Emission Averages, MJ: mega joule, RoW: Rest of World emission averages, HVAC: heating, ventilation and air conditioning.*

**
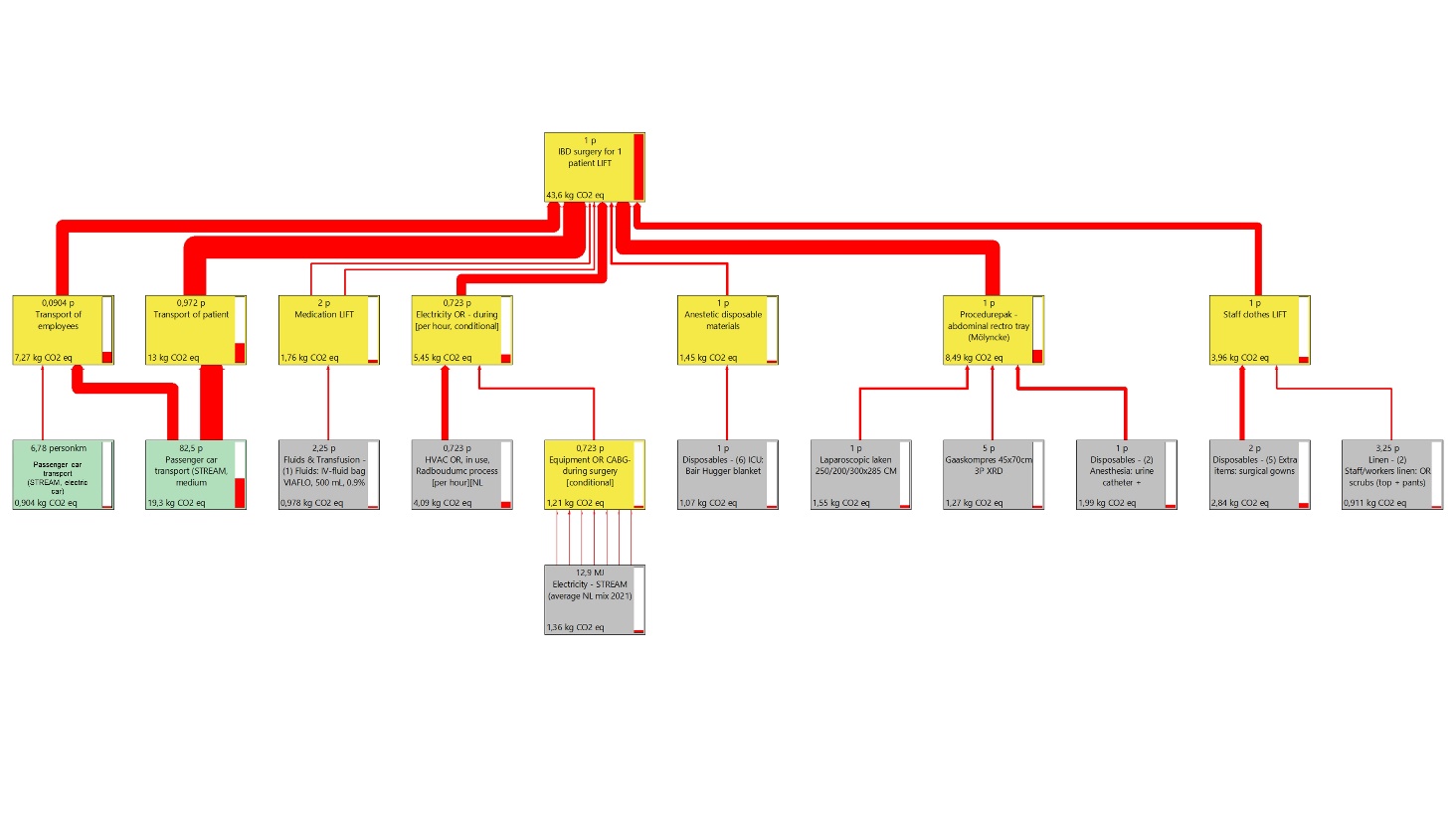
**

**Supplementary Figure 1c** LCA process tree illustrating the impact assessment (1.5% cut-off) expressed in CO2eq of one LIFT procedure in a Crohn’s PAF patient. Thickness of the red lines shows the impact of each input. *Eq: equivalents, GLO: Global Emission Averages, MJ: mega joule, RoW: Rest of World emission averages, HVAC: heating, ventilation and air conditioning.*


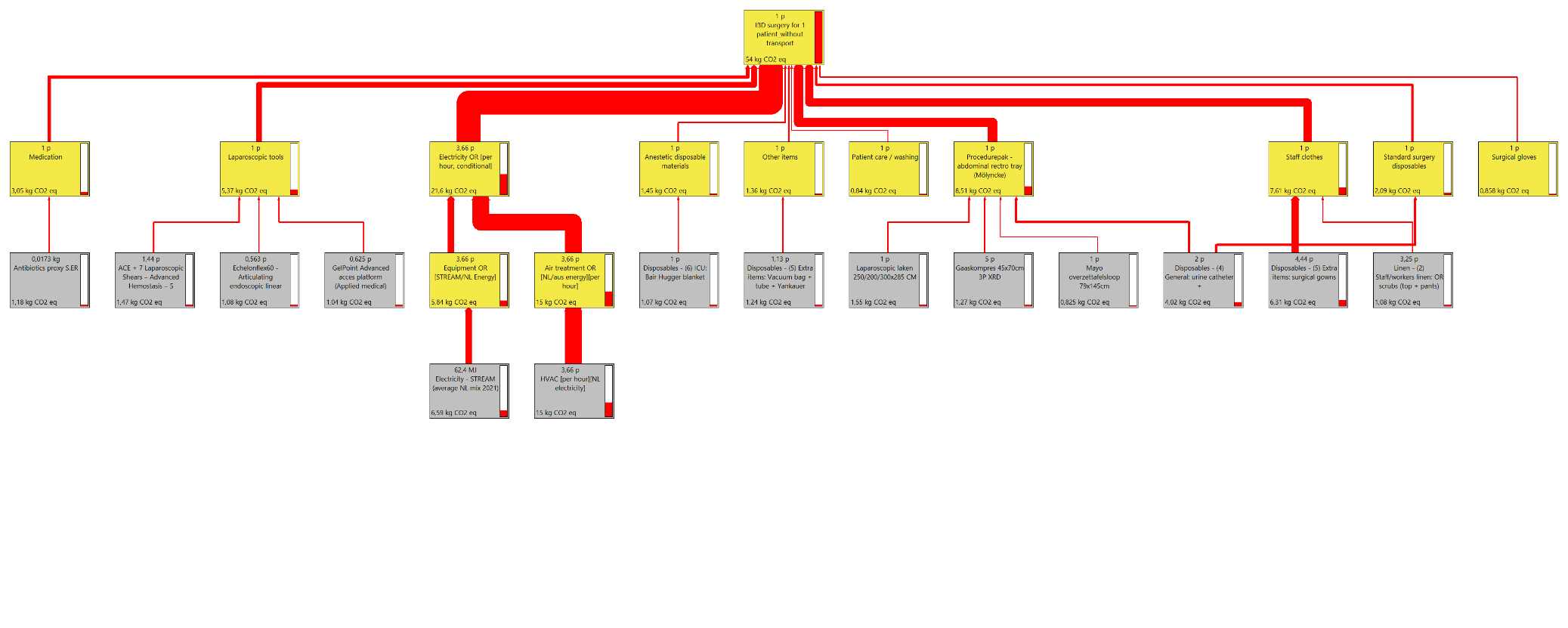


**Supplementary Figure 2a** LCA process tree illustrating the impact assessment (1.5% cut-off) expressed in CO2eq of one laparoscopic ICR for terminal ileitis in CD (without transport of personnel and patients). Thickness of the red lines shows the impact of each input. *Eq: equivalents, GLO: Global Emission Averages, MJ: mega joule, RoW: Rest of World emission averages, HVAC: heating, ventilation and air conditioning.*

**
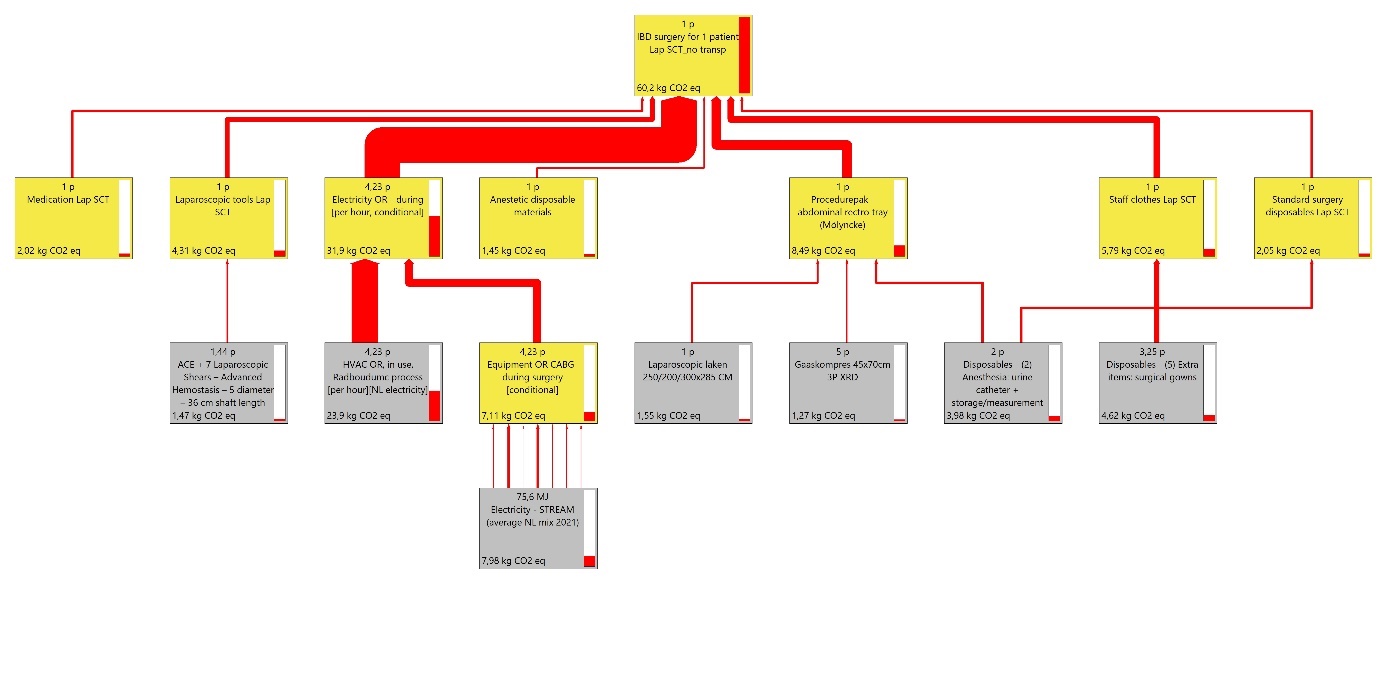
**

**Supplementary Figure 2b** LCA process tree illustrating the impact assessment (1.5% cut-off) expressed in CO2eq of one laparoscopic STC in UC (without transport of personnel and patients). Thickness of the red lines shows the impact of each input. *Eq: equivalents, GLO: Global Emission Averages, MJ: mega joule, RoW: Rest of World emission averages, HVAC: heating, ventilation and air conditioning.*

**
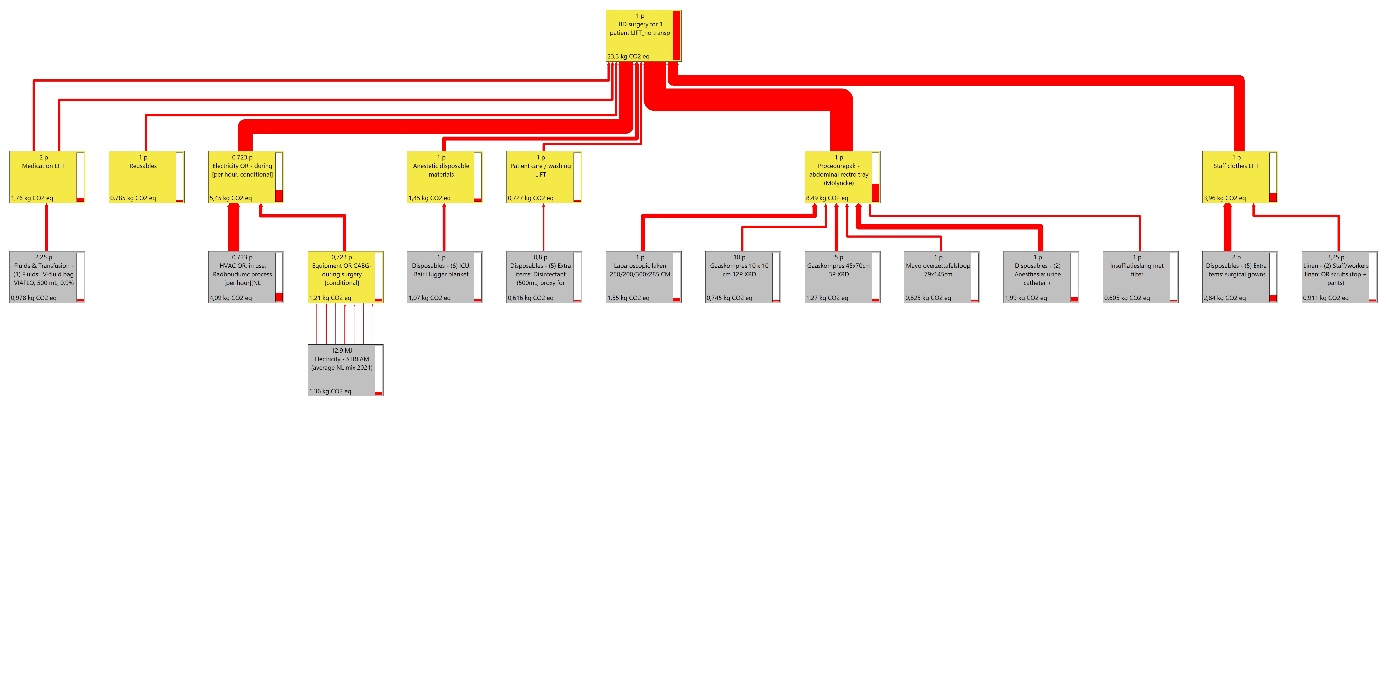
**

**Supplementary Figure 2c** LCA process tree illustrating the impact assessment (1.5% cut-off) expressed in CO2eq of one LIFT procedure in a Crohn’s PAF patient (without transport of personnel and patients). Thickness of the red lines shows the impact of each input. *Eq: equivalents, GLO: Global Emission Averages, MJ: mega joule, RoW: Rest of World emission averages, HVAC: heating, ventilation and air conditioning.*

**
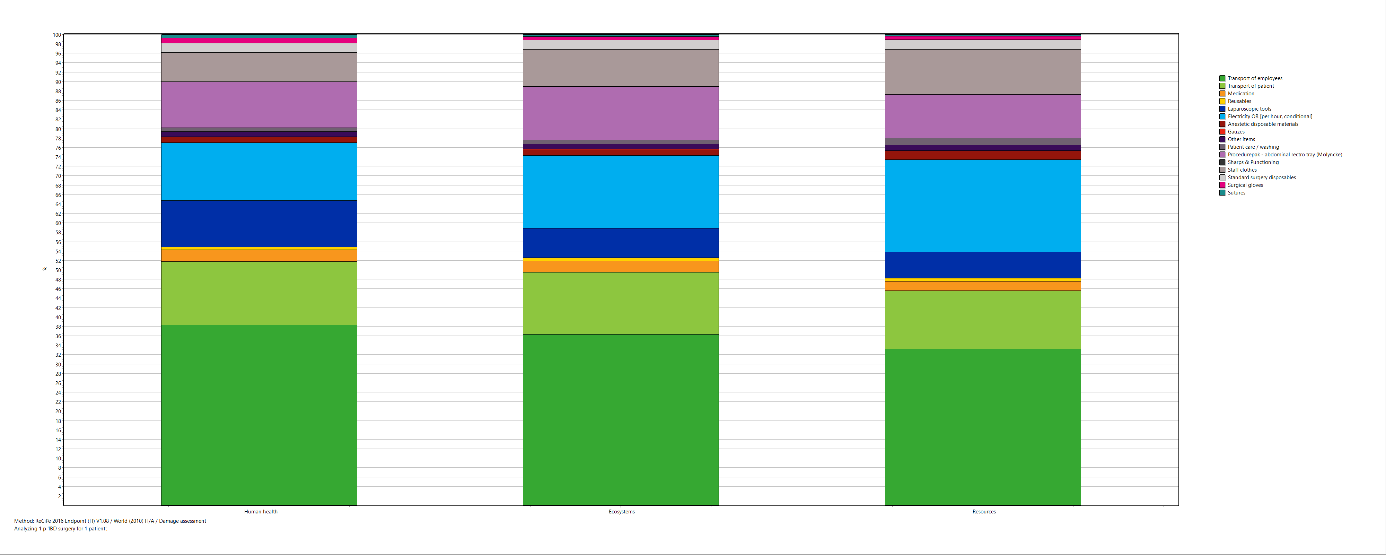
**

**Supplementary Figure 3a** Endpoint analysis of laparoscopic ICR for terminal ileitis in CD patients – damage assessment


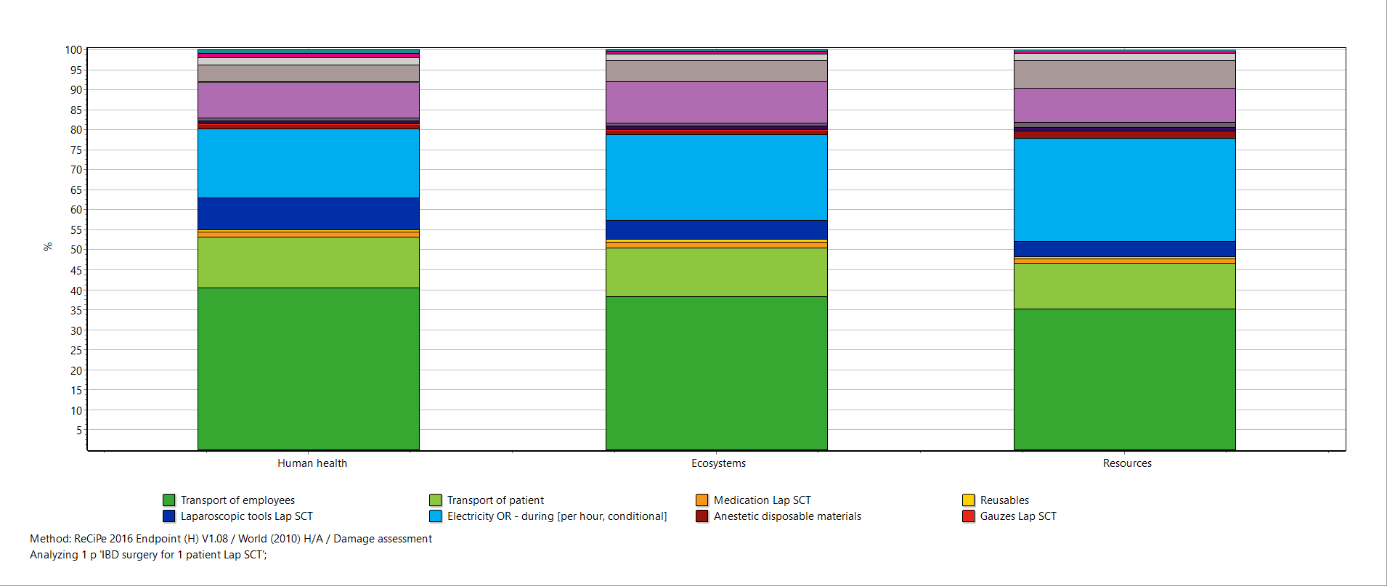


**Supplementary Figure 3b** Endpoint analysis of laparoscopic STC in UC – damage assessment


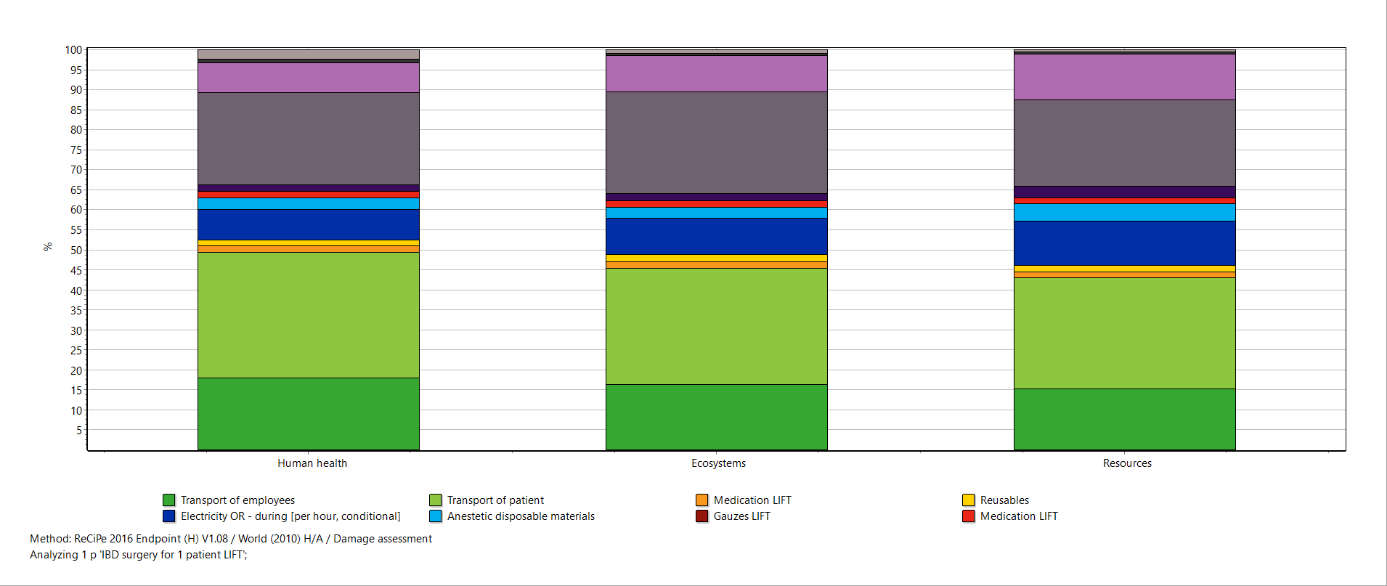


**Supplementary Figure 3c** Endpoint analysis of LIFT procedures in Crohn’s PAF patients – damage assessment
