# Supplementary material for: Carbon footprint of common procedures in inflammatory bowel disease
Source: Tech Coloproctol. 2025 May 30;29(1):127. doi: 10.1007/s10151-025-03123-5 (PMC12125120; doi:10.1007/s10151-025-03123-5)
Supplement: Supplementary file 2 — Supplementary file2 (70 KB) [file 10151_2025_3123_MOESM2_ESM.docx]

**Carbon footprint of common procedures in Inflammatory Bowel Disease**

Drs. Liesbeth Munster^1,2^, MSc Bas van der Zwet^1,2^, dr. Joline de Groof^1,2^, dr. Marco Mundt^3^, dr. Oddeke van Ruler^4^, prof. dr. Geert D’Haens^5^, prof. dr. Willem Bemelman^2^, dr. Christianne Buskens^2^, dr. Marjolijn Duijvestein^6^, dr. Tim Stobernack^7^, dr. Jarmila van der Bilt^1,2^

^1^ Department of Surgery, Flevoziekenhuis, Almere, The Netherlands

^2^ Department of Surgery, Amsterdam UMC (location VUmc), Amsterdam, The Netherlands

^3^ Department of Gastroenterology and Hepatology, Flevoziekenhuis, Almere, The Netherlands

^4^ Department of Surgery, IJsselland Hospital/Erasmus University Medical Center, Rotterdam, The Netherlands

^5^ Department of Gastroenterology and Hepatology, Amsterdam UMC (location VUmc), Amsterdam, The Netherlands

^6^ Department of Gastroenterology and Hepatology, Radboud University Medical Center, Nijmegen, The Netherlands

^7^ Department of Intensive Care Medicine, Radboud University Medical Center, Nijmegen, The Netherlands

Correspondence to:

Prof. dr. W.A. Bemelman

Amsterdam UMC, location VUmc, Department of Surgery

De Boelelaan 1117, 1081 HV Amsterdam, The Netherlands

Email: w.a.bemelman@amsterdamumc.nl

Telephone: +31-20-4441100

Correspondence during review process

Drs. L.J. Munster

Email: l.munster@amsterdamumc.nl

Running title: Surgical carbon footprint in IBD

Article type: Cross-sectional study

Funding: none, original article

**Abstract**

*Background:*The aim of this study was to assess the environmental impact, primarily the carbon footprint of the most common procedures in Inflammatory Bowel Disease (IBD).

*Methods*:In this study, all processes and products used during a total of eight laparoscopic ileocecal resection (ICR) in Crohn’s disease (CD) patients, eight laparoscopic subtotal colectomies (STC) for ulcerative colitis (UC), and eight ligation of the intersphincteric fistula tract (LIFT) procedures in Crohn’s perianal fistula (PAF) patients (all in adults ≥16 years) between March 2023 and May 2024 were collected. A Life Cycle Assessment (LCA) was conducted and mean CO2-emission rates were calculated, the major contributors (“hotspots”) were determined and midpoint/endpoint-analysis was performed.

*Results*:The mean total carbon footprints of laparoscopic ICR, STC and LIFT were respectively 104kg, 116kg, and 43.6kg CO2eq, equaling one-way trips by airplane from Amsterdam to Paris, to Manchester, and to Düsseldorf respectively. Main contributors in laparoscopic ICR and STC were transport of employees/patients (48%/49% respectively), energy use in theatre (21%/27% respectively), and the use of surgical equipment (14%/17% respectively). In LIFT procedures, transport of employees/patients accounted for 47% of total emission rates, followed by the use of surgical equipment (28%), and electricity use in theatre (13%). Besides the impact on global warming, significant impact on fine particulate matter formation, land use, terrestrial acidification and fossil resource scarcity was identified. Endpoint-analysis showed an amount of disability-adjusted life years (DALY’s) of approximately two hours of health damage per laparoscopic ICR/STC, and 47 minutes per LIFT.
*Conclusions*:The carbon footprint of three commonly performed IBD-surgeries is mainly determined by transportation of patients/health care personnel, followed by electricity and material use. The latter two vary with the complexity of the surgeries. IBD-surgeons should focus on minimize energy resources and the use of standard surgical materials. Also, employees should be encouraged to traveling by foot/bicycle/public transport/carpooling/electric car.

**Keywords:** Crohn’s disease; laparoscopic ileocecal resection; laparoscopic subtotal colectomy; LIFT; carbon footprint; sustainability.

**Key Summary**

1. Summary of the established knowledge on this subject
   - The healthcare system is (partly) responsible for generation of so called greenhouse gases (GHG).
   - Within hospitals, surgical theaters are held responsible for the production of a significant amount of direct (e.g. gas, electricity) as well as indirect (e.g. the use of many supplies or production of waste) GHG.
   - There is a significantly growing interest in sustainable healthcare that minimizes the carbon footprint.
2. New findings of this study
   - In this study the environmental impact, primarily the carbon footprint, of laparoscopic ICR for terminal ileitis in CD patients, laparoscopic STC in UC patients, and LIFT procedure in Crohn’s PAF patients was assessed.
   - The carbon footprint of these three commonly performed surgeries in IBD is mainly determined by transportation of patients and health care personnel, followed by electricity and material use. The latter two vary with the complexity of the surgeries.
   - IBD-surgeons should focus on minimize energy resources and the use of standard surgical materials.
   - Employees should be encouraged to traveling by foot, bicycle, public transport, carpooling or electric car.

**Introduction**

According to the World Health Organization (WHO), climate change, including global warming, is one of the largest healthcare threats of the 21^st^ century due to the generation of so-called greenhouse gases (GHG), such as carbon dioxide (CO2) [1]. As a paradox, human activities and the healthcare system itself are (partly) responsible for generation of those GHG [2,3]. Healthcare emissions are estimated to contribute to a combined 5% of global net GHG emissions [2,4], which is comparable in the UK with an estimated amount of 3-4%, and even higher in Australia (7%) [5] and the USA (10%) [6]. Although the actual amount of CO2 emission in healthcare is still a matter of debate, with varying numbers over time in all different countries, there is a significantly growing interest in sustainable healthcare that minimizes the carbon footprint [7,8,9].

Within hospitals, surgical theaters are held responsible for the production of a significant amount of direct (e.g. gas, electricity) as well as indirect (e.g. the use of many supplies or production of waste) GHG [4,10,11]. Studies into the CO2 footprint of several surgical procedures (e.g. laparoscopic right hemicolectomy, cataract surgeries, total knee replacements or surgery in general) [12,13,14,15,16] have enabled surgeons to reduce (in)direct emission rates by development of surgery-specific CO2 reduction strategies [16]. Moreover, nowadays, in clinical decision making between different treatment options with similar clinical results, the environmental impact is considered a new but growing outcome parameter alongside quality of life (QoL) and costs-effectiveness to tailor healthcare decisions. This may even be more relevant in the context of chronic disease, such as Inflammatory Bowel Disease (IBD), where the cumulative environmental impact of treatments can be substantial. Recently, the LIR!C trial showed that laparoscopic ileocecal resection (ICR) can be considered an alternative and cost-effective treatment option to infliximab treatment in a subgroup of Crohn’s disease (CD) patients (presenting with limited ileocecal CD and failing on conventional therapy) [17,18,19]. No data on the GHG production in CD patients undergoing laparoscopic ICR are to the best of our knowledge available, which is similar to other frequently performed IBD related surgeries, such as laparoscopic subtotal colectomy (STC) in ulcerative colitis (UC) patients, and ligation of the intersphincteric fistula tract (LIFT) procedure in Crohn’s perianal fistula (PAF) patients. The aim of this study was to assess the environmental impact, primarily the carbon footprint, of laparoscopic ICR for terminal ileitis in CD patients, laparoscopic STC in UC patients, and LIFT procedure in Crohn’s PAF patients, and to determine which surgery-specific components contribute the most in order to develop surgery-specific carbon reduction strategies.

**Materials and methods**

*Study design and population*

This is a prospective, observational, multicentre cross-sectional study evaluating the CO2 generation of laparoscopic ICR for terminal ileitis in CD patients, laparoscopic STC in UC patients, and LIFT procedures in Crohn’s PAF patients. This study was conducted in two non-academic teaching hospitals (Flevoziekenhuis, Almere, The Netherlands and IJsselland ziekenhuis, Rotterdam, The Netherlands) between March 2023 and May 2024. According to previous studies [12,13,14,15,16] and the exploratory nature of this study, eight laparoscopic ICR, eight laparoscopic STC, and eight LIFT procedures (n=24 surgeries in total) were considered as a minimal sample size in this study to provide a reproducible calculation and general overview of the CO2(-equivalent) generation as a result of these surgeries.

This study was conducted according to the ‘Strengthening the Reporting of Observational Studies in Epidemiology’ (STROBE) guidelines for cross-sectional studies.

*Inclusion and exclusion criteria*

All processes and products used during a total of eight laparoscopic ICR for terminal ileitis, eight laparoscopic STC in UC patients, and eight LIFT procedures in Crohn’s PAF patients (all in adult patients ≥16 years) between March 2023 and May 2024 were prospectively observed by two researchers (LM and BZ) in both participating centres. Exclusion criterium was the conversion of a laparoscopic ICR or laparoscopic STC into an open resection.

*Ethical approval*

This study did not fall within the scope of the Medical Research Involving Human Subjects Act (WMO), since patients were only indirectly involved in this study. Therefore, ethical approval by an ethics committee (METC) was not obtained. However, this project was conducted according to the principles of the Declaration of Helsinki (64^th^ WMA General Assembly, Fortaleza, Brazil, October 2013) and according to the General Data Protection Regulation (GDPR).

*Life cycle assessment (LCA)*

In healthcare, LCA’s are used as a tool to evaluate and compare the environmental

impact, including carbon footprint, of all products and processes. This methodology has experienced a significant growth in the past decade and has been applied to similar processes of healthcare [20,21,22]. LCA’s are internationally standardized approaches enabling the quantification of materials and energy input and output, throughout the lifecycle of a predefined study object. LCA’s also allow to assess the environmental impact of regional and global resources to evaluate its depletion and environmental degradation, thereby representing the environmental implications of human activities [23]. Typically, LCA’s involve four phases. In the first phase, the scope (including system boundaries) and eventual goals of the LCA are defined. Secondly, an inventory analysis for all processes in the life cycle, including all relevant (technical) in-/outputs, emissions and the use of resources, is conducted. The inventory of the current study was created by using the EcoInvent database (version 3.9) as described at https://ecoinvent.org/the-ecoinvent-database (*Accessed February 21, 2024*). In the third phase of the LCA the impact of all (grouped) resources and emissions are evaluated and quantified to conduct comparable groups. In the last phase, results of the environmental impact findings are interpreted [23]. In this way, potential so called ‘hot spots’ according the environmental impact can be identified [23,24]. The SimaPro software package (version 9.5.0.2), including the ReCipe 2016 Endpoint (h) V1.08 / World (2010) H/A method, was used to conduct midpoint as well as endpoint analysis in this LCA [25,26]. Midpoint analyses are primarily focused on separate environmental details (e.g. water consumption or global warming), whereas endpoint analyses are focused on the general effect and damage on three aggregation levels (also known as ‘areas of protection’): the impact on our ecosystem (in extinct species per year), human health (in DALY’s: disability-adjusted life years) and resources (in US-dollars) [25,26], in this study as a result of one surgery. DALY’s represent the comprehensive disease burden in a population, indicative for the total number of years lost due to disability, premature death or poor health conditions [27].

*System boundary and data collection*

As mentioned above, the first phase of the LCA included a predefined system boundary (Figure 1). This system boundary included all specific (peri-)operative processes of a laparoscopic ICR, a laparoscopic STC, and a LIFT procedure, including materials (e.g. disposables and reusables), energy (e.g. power usage of devices and light, cooling/heating), medication/anaesthetics (e.g. sedative medication, analgesics, antibiotics or anticoagulants), waste (pre, intra- and postoperative, including the type of waste) and cleaning/washing (e.g. cleaning of the theatre, sterilization of reusables and washing of surgery clothing). The system boundary served as reference in the data collection process. In the second phase, an inventory list based on the system boundary was created, including all relevant data that needed to be observed during all surgeries by two researchers (LM and BZ), and checked by LM. This input list covered both fixed and variable elements and all stages in the LCA (production and distribution of all products) were considered. For all items used during a laparoscopic ICR, a laparoscopic STC, and a LIFT procedure, the following data were recorded: materials, weight/volume, manufacturing place, supplier location, distributor location, distance from distributor to the hospital, transportation means and prices. Modes of transportation were recorded and travelling distances from and to the hospital of both staff and patients were calculated by the use of Google Maps (https://www.google.nl/maps). Afterwards, those modes and distances were converted into CO2 equivalents (kg) by the use of SimaPro and the ReCipe method as mentioned above. As employees are expected to be at work, regardless whether a laparoscopic ICR, STC or LIFT is being scheduled, and mobility emission rates could vary within hospitals depending on urban or rural localization and hence accessibility, and in order to improve comparability (e.g. to compare the environmental impact of treatments with similar clinical results) we separately analyzed the environmental impact of the procedure itself, i.e. without transport of personnel and patient.

Waste consumption data were derived from proxy data from Radboud University Medical Center (Radboudumc), The Netherlands, as well as missing data (or reported if not possible). Energy consumption in theatre was measured using a standard in hospital energy gauge (kilowatt per hour). Food and drinks as consumed by the patients and staff were not considered. Similarly, details on construction of the hospital and the production of machines were excluded.

In order to facilitate comparable and straightforward CO2 equivalent emission rates, a comparison was made between the outcomes in this study and travelling distances by airplane or car, using conversion factors as provided by https://www.co2emissiefactoren.nl/ (Well to Wheel conversion factor of 0.234 kilograms per kilometer for short distance flights, and 0.193 kilograms per kilometer for distances by car).

**Results**

All processes and products involved in a total of eight laparoscopic ICR’s for terminal ileitis in CD patients, eight laparoscopic STC in UC patients, and eight LIFT procedures in Crohn’s PAF patients were analyzed. The mean total carbon footprint was 104 kg CO2eq in laparoscopic ICR for terminal ileitis for CD, 116 kg CO2eq in laparoscopic STC for UC, and 43.6 kg CO2eq in a LIFT procedure in Crohn’s PAF patients (including transport of staff and patients), which equals one-way trips by airplane from Amsterdam (The Netherlands) to Paris (France, 430 km by air), to Manchester (England, 494 km by air), and to Düsseldorf (Germany, 182 km by air) respectively. Figure 2 shows Tree maps of the LCA of ICR for terminal ileitis in CD patients, laparoscopic STC in UC patients, and LIFT procedures in Crohn’s PAF patients, indicating all so called “hotspots” in the carbon footprint of one single surgery (including transport of personnel and patients). Supplementary Figure 1 shows a complete overview of the entire LCA in process trees (including transport of personnel and patients, 1.5% cut-off). The environmental impact analysis showed several main contributors. In laparoscopic ICR these were transport of employees (35%), transport of patients (13%), electricity in theatre (21%), medication (3%), laparoscopic tools (5%), and a combination of standard surgery items (including surgical drapes/equipment covers, staff clothes and standard surgery disposables, 17% combined). The LCA of transport of employees and patients accounted for the largest share of the total carbon footprint (50.2 kg CO2eq, 48% combined), followed by energy use in theatre (21.6 kg CO2eq, 21%).

In laparoscopic STC main contributors were quite similar as compared to laparoscopic ICR, including transport of employees (37%), transport of patients (12%), electricity in theatre (27%), laparoscopic tools (4%), a combination of standard surgery items (including surgical drapes/equipment covers, staff clothes and standard surgery disposables, 14% combined), medication (2%), and anesthetic disposables (1%). In this type of surgery, the LCA of transport of employees and patients also accounted for the largest share of the total carbon footprint, and total CO2eq emission rates were slightly higher as compared to laparoscopic ICR (56.1kg CO2eq, 49% combined), followed by energy use in theatre (31.9kg CO2eq, 27%. The difference between laparoscopic ICR and STC can mainly be explained by difference in duration of the surgery, and consequently the use of theatre in general, which was longer in STC.

In LIFT procedures for Crohn’s PAF, transport of patients was a larger contributor (30%) as compared to transport of employees (17%), which was the other way around in laparoscopic ICR and STC. Other main contributors were a combination of standard surgery items (including surgical drapes/equipment covers, staff clothes and standard surgery disposables, 28% combined), electricity use in theatre (13%), anesthetic disposables (3%), medication (2%), and reusables (2%).

The mean total carbon footprint of a laparoscopic ICR for terminal ileitis without transport of personnel and patients, was 54.0 kg CO2eq, equaling a one-way trip by car from Amsterdam (The Netherlands) to Cologne (Germany, 264 km by car). In laparoscopic STC this was 60.2 kg CO2eq, equaling a one-way trip by car from Amsterdam to Luxembourg (Luxembourg, 361 km by car), and in LIFT procedures this was 23.3 kg CO2eq, equaling a trip from Amsterdam to Eindhoven (The Netherlands, 125 km by car). When excluding transport of employees and patients, it was showed that main carbon footprint contributors were electricity use in theatre, including air treatment (heating, ventilation and air conditioning) and equipment, with a mean estimated 21.6 kg, 31.9 kg, and 5.5 kg CO2eq emissions in laparoscopic ICR, STC and LIFT procedures respectively, which accounted for 40%, 53%, and 24% of total CO2eq emissions respectively. These emission rates were directly correlated with the duration and complexity of surgery, and consequently the use of theatre in general, since long and complex procedures require more theatre time and material.

In LIFT procedures, the use of surgical equipment accounted for an even larger share with a combined 12.5kg CO2eq emission rate, which accounted for 54% of the total CO2eq emission rates of a LIFT procedure solely (without transport of employees and patients). LCA’s, illustrating the impact assessment of all three procedures without transport of personnel and patients, are presented in Supplementary Figure 2.

*Midpoint analysis*

Figure 3 illustrates the midpoint analyses showing the impact of laparoscopic ICR for terminal ileitis in CD, laparoscopic STC in UC, and LIFT procedures in Crohn’s PAF patients on several specific environmental factors (whereas endpoint analyses provide a more general overview). As mentioned above, transport of employees and patients, as well as energy use in theatre, and the use of surgical equipment are major contributors to CO2eq emissions in all three surgeries. Besides the impact of those factors on global warming (86%, 90%, and 88% respectively of the total impact on global warming), significant impact on fine particulate matter formation (82%, 85%, and 89% respectively% of the total impact on fine particulate matter formation), land use (in all three surgeries 94% of the total impact on land use), terrestrial acidification (81%, 85%, and 90% respectively of the total terrestrial acidification) and fossil resource scarcity (86%, 90%, and 87% respectively of the total fossil resource scarcity) was identified.

*Endpoint analysis*

Endpoint analyses of all three surgeries are shown in Table 1. The amount of DALY’s (0.000215 and 0.000235 for laparoscopic ICR and STC respectively) are equal to approximately two hours of health damage in one person worldwide per single laparoscopic ICR or laparoscopic STC. For a singe LIFT procedure (with DALY 0.000090) this equals approximately 47 minutes in one person worldwide. An overview of the damage assessment per category is presented in Supplementary Figure 3.

**Discussion**

This study showed a mean total carbon footprint of 104 kg CO2eq, 116 kg CO2eq, and 43.6kg CO2eq in laparoscopic ICR for CD, laparoscopic STC for UC, and LIFT procedures in Crohn’s PAF patients respectively. This equals one-way trips by airplane from Amsterdam to Paris, to Manchester, and to Düsseldorf respectively. Remarkably, the main contributors in laparoscopic ICR and STC were transport of health care personnel and patients (48% and 49% respectively), and energy use in theatre (21% and 27% respectively). In LIFT procedures, the transport of health care personnel and patients accounted for 47% of total emission rates, electricity use in theatre for 13%, and also the use of surgical equipment accounted for a large share (28%). This can be explained by the fact that transportation of patients and medical personal is relatively constant, while electricity and material use depend on the type of procedure. Long and complex procedures require more theatre time and material.

The healthcare system, in particular surgical care, is being held responsible for a fair amount of generation of GHG, including CO2. Surgical theaters expend three to six times more energy than other departments in hospitals [28,29] and surgical waste is an important topic of interest in the field of GHG emissions [30]. It is therefore inevitable that surgical associations, as for example the American College of Surgeons, started to raise more awareness on this topic [31]. Introducing the carbon footprint as a new decisive outcome parameter incentivizes the adoption of more sustainable healthcare practices. It encourages the development and utilization of (medical) technologies, procedures, and treatments that minimize environmental harm without compromising patient care (e.g. minimizing the use of redundant (sedative) medication/items in theatre, the use of renewable energy sources or telemonitoring, if possible, to reduce transport movements) [31].

To our knowledge this is the first study that assessed the detailed environmental impact of frequently performed IBD-related surgeries as laparoscopic ICR, laparoscopic STC and LIFT. Although we do acknowledge that it is challenging to compare these surgeries with each other, the carbon emission rates as a result of laparoscopic ICR, STC and LIFT procedures were calculated by an exhaustive LCA, which could serve as a benchmark for future studies and surgery specific reduction strategies. Comparing other surgical procedures, as indicated in a systematic review of Rizan et al., showing that the carbon footprint of a single surgery ranged from 6 to 814 kg CO2eq, the current study showed that the carbon footprint of a laparoscopic ICR, STC and LIFT were at the lower end of the spectrum [12]. Taylor et al. showed even lower emission rates in laparoscopic right hemicolectomy (22.21 kg CO2eq), which could be explained by the fact that they excluded anesthetics and perioperative materials [16]. Transanal mesorectal excision (TME) for cancer (408,6 kg CO2eq) and robotic hysterectomy (814 kg CO2eq) are examples at the upper end [12,13,14,15,16,32,33]. However, it should be kept in mind that comparing (surgical) GHG-emission studies directly is difficult due to heterogeneity and methodological differences between all studies (e.g. various system boundaries) [12,16]. Also, it should be kept in mind that healthcare protocols potentially differ per hospital or region. As an example, in the current study, it was remarkable that the included hospitals only used total intravenous anesthesia (TIVA) as part of their so called ‘Green Team initiatives’ as compared to other (mostly non-academic) hospitals/studies that often use anesthetic gases during surgery. The latter is inherent to higher healthcare emission rates.

While most studies reported on the carbon footprint (alias global warming) as a result of a specific surgery only, we also provide detailed insight into several other important aspects of the environmental impact, including the impact on fine particulate matter formation, land use, terrestrial acidification and fossil resource scarcity. Moreover, endpoint analyses showed that laparoscopic ICR and STC resulted in approximately two hours of health damage per laparoscopic ICR and STC (expressed as DALY’s), and 47 minutes in LIFT procedures, which is indicative for the total number of hours lost due to disability, premature death or poor health conditions in one person worldwide [27], paradoxically in this case mainly affecting people from lower income countries (since the impact of climate change on health is assumed to be much higher in low versus high income countries) from where most resources were derived [7,8,9].

Although awareness is increasing and a variety of initiatives, e.g. so called “Green Teams”, emerge in order to reduce emission rates in theatres according to the “5 R principle” (Reduce, Reuse, Recycle, Rethink and Research, e.g. energy savings, the use of less instruments and textiles, telehealth visits, the use of TIVA instead of anesthetic gases) [13,34], there is still considerable room for improvement. Based on this study, surgeons (and anesthesiologists) should focus on minimizing energy resources and the use of standard surgical products (including surgical drapes/equipment covers, staff clothes and standard surgery disposables), without compromising safety and outcomes. Especially in less invasive surgeries as a LIFT procedure (with proportionally shorter time in theatre), it should be kept in mind that the use of surgical equipment account for the largest share in CO2eq emission rates, and that the use of equipment should be reduced if possible. In line with this, all attending healthcare employees should encourage the use of sustainable alternatives if possible (e.g. reusable surgical caps instead of disposables, although controversially it should be kept in mind that sterilization processes also account for its share in total CO2eq emission rates). In order to reduce electricity use in theatre, the attending surgeon and anesthesiologist should always strive to the shortest time as possible in theatre. We do feel that the importance of the healthcare footprint reduction strategies should be addressed to all surgeons and should be applied during training of young, new surgeons. Last, but definitely not least, this study should empower healthcare professionals and decision-makers to reduce the healthcare footprint simply by encouraging employees (and if possible patients) to traveling by foot, bicycle, public transport, carpooling or electric car.

This study has several limitations inherently to its design. We did not take into account the use of hospital beds, material and energy expenditure at the ward, which are important for the procedures where there is an admission necessarily. Therefore, in order to improve comparability with other (pharmaceutical) treatment options (of whom data on GHG-emission are currently often lacking), it would be recommended to also account for amongst others length of hospital stay and all other equipment used during hospital stay, complications, reinterventions or admission to the Intensive Care Unit (ICU), including transport of not only all medical stakeholders but also of all visitors. More detailed data on the environmental impact of different treatment modalities in IBD, but also in chronic disease in general, may contribute to sustainable clinical decision making in the future, particularly if surgery proved to be an alternative to medical management. Lastly, it should be kept in mind that the current study was conducted in rural hospital settings, which may have led to an overrepresentation of travel emissions of patients as well as employees as compared to other urban hospital settings.

In conclusion, the carbon footprint of three commonly performed surgeries in IBD is mainly determined by transportation of patients and health care personnel, followed by electricity and material use. The latter two vary with the complexity of the surgeries. IBD-surgeons should focus on minimize energy resources and the use of standard surgical materials. Also, employees should be encouraged to traveling by foot, bicycle, public transport, carpooling or electric car.

**Funding**

This study was not funded (original article).

**Data availability statement**

All authors of this manuscript confirm that the data supporting the findings of this study are available within the manuscript. Supplementary details (including a complete overview of the inventory list) are available upon reasonable request.

**Disclosures**

Prof. Geert D’Haens has served as advisor for AbbVie, Ablynx, Alimentiv, Amgen, AM Pharma, Biogen, Bristol Meiers Squibb, Boehringer Ingelheim, Celgene/Receptos, Celltrion, Cosmo, Covidien/Medtronic, Ferring, Dr Falk Pharma, Eli Lilly, Engene, Galapagos, Genentech/Roche, Gilead, GlaxoSmithKline, Immunic, Johnson and Johnson, Lamepro, Lument, Mitsubishi Pharma, Merck Sharp Dome, Mundipharma, Nextbiotics, Novo Nordisk, Otsuka, Pfizer, Polpharm, Prometheus Laboratories/Nestlé, Procise Diagnostics, Protagonist, Salix, Samsung Bioepis, Sandoz, Setpoint, Shire, Takeda, Tigenix, Tillotts, Topivert, Versant and Vifor; and received speaker fees from AbbVie, Biogen, Ferring, Johnson and Johnson, Merck Sharp Dome, Mundipharma, Norgine, Pfizer, Samsung Bioepis, Shire, Takeda, Tillotts, and Vifor. Prof. Willem Bemelman has received research funding from VIFOR, has a 4.9% share in Semiflex, received speakers fee from Galapagos and is a consultant for Braun and Olympus. Dr. Christianne Buskens received an unrestricted grant from Boehringer Ingelheim and Roche. She has received consultancy/speakers fees from Tillotts, MSD, Takeda, Janssen, Galapagos. Dr. Marjolijn Duijvestein received Speaking fee from Bristol Meyers Squibb, Takeda, Galapagos. Served in an advisory board for Abbvie, Bristol Meyers Squibb, Celltrion, Galapagos, Janssen, Takeda. Received Grant/Research support from Pfizer, Bristol Meyers Squibb, Galapagos and Janssen. Dr. Oddeke van Ruler has served as invited speaker for Janssen-Cilag; and has received a research grant from Takeda and Janssen, outside the submitted work. Dr. Tim Stobernack has received research funding from ZonMw (file number 80-86800-98-112). Drs. Liesbeth Munster, MSc. Bas van der Zwet, dr. Joline de Groof, dr. Marco Mundt and dr. Jarmila van der Bilt have no conflicts of interest or financial ties to disclose.

**References**

1. Watts N, Amann M, Arnell N, Ayeb-Karlsson S, Beagley J, Belesova K, Boykoff M, Byass P, Cai W, Campbell-Lendrum D, Capstick S, Chambers J, Coleman S, Dalin C, Daly M, Dasandi N, Dasgupta S, Davies M, Di Napoli C, Dominguez-Salas P, Drummond P, Dubrow R, Ebi KL, Eckelman M, Ekins P, Escobar LE, Georgeson L, Golder S, Grace D, Graham H, Haggar P, Hamilton I, Hartinger S, Hess J, Hsu SC, Hughes N, Jankin Mikhaylov S, Jimenez MP, Kelman I, Kennard H, Kiesewetter G, Kinney PL, Kjellstrom T, Kniveton D, Lampard P, Lemke B, Liu Y, Liu Z, Lott M, Lowe R, Martinez-Urtaza J, Maslin M, McAllister L, McGushin A, McMichael C, Milner J, Moradi-Lakeh M, Morrissey K, Munzert S, Murray KA, Neville T, Nilsson M, Sewe MO, Oreszczyn T, Otto M, Owfi F, Pearman O, Pencheon D, Quinn R, Rabbaniha M, Robinson E, Rocklöv J, Romanello M, Semenza JC, Sherman J, Shi L, Springmann M, Tabatabaei M, Taylor J, Triñanes J, Shumake-Guillemot J, Vu B, Wilkinson P, Winning M, Gong P, Montgomery H, Costello A. (2021) The 2020 report of The Lancet Countdown on health and climate change: responding to converging crises. Lancet. DOI: 10.1016/S0140-6736(20)32290-X. Dec 2 2020.
2. Schneider SH. (1989) The greenhouse effect: science and policy. Science. DOI: 10.1126/science.243.4892.771, Feb 10, 1989
3. Eckelman MJ, Sherman J (2016) Environmental Impacts of the U.S. Health Care System and Effects on Public Health. PLoS ONE, <https://doi.org/10.1371/journal.pone.0157014>
4. MacNeill AJ, Lillywhite R, Brown CJ. (2017) The impact of surgery on global climate: a carbon footprinting study of operating theatres in three health systems. Lancet Planet Health. 1:e381–8.
5. Malik A, Lenzen M, McAlister S, McGain F. (2018) The carbon footprint of Australian health care. Lancet Planet Health. DOI: 10.1016/S2542-5196(17)30180-8. Jan 9,2018
6. Chung JW, Meltzer DO. (2009) Estimate of the carbon footprint of the US health care sector. JAMA. DOI: 10.1001/jama. Oct 16, 2009.
7. Lenzen M, Malik A, Li M, Fry J, Weisz H, Pichler PP, Chaves LSM, Capon A, Pencheon D. (2020) The environmental footprint of health care: a global assessment. Lancet Planet Health. 1:e271–9.
8. Romanello M, Di Napoli C, Drummond P, Green C, Kennard H, Lampard P, et al. The 2022 report of the Lancet Countdown on health and climate change: health at the mercy of fossil fuels. Lancet. 2022 Nov 5;400(10363):16
9. Blom IM, Eissa M, Mattijsen JC, Sana H, Haines A, Whitmee S. Effectiveness of greenhouse gas mitigation intervention for health-care systems: a systematic review. Bull World Health Organ. 2024 Mar 1;102(3):159-175B. doi: 10.2471/BLT.23.290464. Epub 2023 Jan 31. PMID: 38420573; PMCID: PMC10898283.
10. McGain F, Jarosz KM, Nguyen MN, Bates S, O'Shea CJ. (2015) Auditing Operating Room Recycling: A Management Case Report. A A Case Rep. DOI: 10.1213/XAA.0000000000000097. Aug 1, 2015
11. Kubicki MA, McGain F, O'Shea CJ, Bates S. (2015) Auditing an intensive care unit recycling program. Crit Care Resusc. 17:135-40.
12. Rizan C, Steinbach I, Nicholson R, Lillywhite R, Reed M, Bhutta MF. (2020) The Carbon Footprint of Surgical Operations: A Systematic Review. Ann Surg. DOI: 10.1097/SLA.0000000000003951.
13. Ferrero A, Thouvenin R, Hoogewoud F, Marcireau I, Offret O, Louison P, Monnet D, Brézin AP. (2022) The carbon footprint of cataract surgery in a French University Hospital. J Fr Ophtalmol. 2022 DOI: 10.1016/j.jfo.2021.08.004. Nov 22 2022.
14. McGain, F., Sheridan, N., Wickramarachchi, K., Yates, S., Chan, B. & McAlister, S. (2021). Carbon Footprint of General, Regional, and Combined Anesthesia for Total Knee Replacements. Anestesiology DOI:10.1097/ALN.0000000000003967
15. Morris DS, Wright T, Somner JE, Connor A. (2013) The carbon footprint of cataract surgery. Eye (Lond). DOI 10.1038/eye.2013.9. Feb 22,2013.
16. Taylor AS, Au S, Krivankova B, Asanai K, Manimaran N, Carbon footprint of laparoscopic right hemicolectomy, British Journal of Surgery, Volume 111, Issue 1, January 2024, znad422, https://doi.org/10.1093/bjs/znad422
17. Ponsioen CY, de Groof EJ, Eshuis EJ, Gardenbroek TJ, Bossuyt PMM, Hart A, Warusavitarne J, Buskens CJ, van Bodegraven AA, Brink MA, Consten ECJ, van Wagensveld BA, Rijk MCM, Crolla RMPH, Noomen CG, Houdijk APJ, Mallant RC, Boom M, Marsman WA, Stockmann HB, Mol B, de Groof AJ, Stokkers PC, D'Haens GR, Bemelman WA; LIR!C study group. (2017) Laparoscopic ileocaecal resection versus infliximab for terminal ileitis in Crohn's disease: a randomised controlled, open-label, multicentre trial. Lancet Gastroenterol Hepatol. DOI: 10.1016/S2468-1253(17)30248-0. Aug 31,2017. Erratum in: Lancet Gastroenterol Hepatol. 2017 Nov;2(11):e7.
18. Stevens TW, Haasnoot ML, D'Haens GR, Buskens CJ, de Groof EJ, Eshuis EJ, Gardenbroek TJ, Mol B, Stokkers PCF, Bemelman WA, Ponsioen CY; LIR!C study group. (2020) Laparoscopic ileocaecal resection versus infliximab for terminal ileitis in Crohn's disease: retrospective long-term follow-up of the LIR!C trial. Lancet Gastroenterol Hepatol. DOI: 10.1016/S2468-1253(20)30117-5. Jun 30,2020.
19. de Groof EJ, Stevens TW, Eshuis EJ, Gardenbroek TJ, Bosmans JE, van Dongen JM, Mol B, Buskens CJ, Stokkers PCF, Hart A, D'Haens GR, Bemelman WA, Ponsioen CY; LIR!C study group. (2019) Cost-effectiveness of laparoscopic ileocaecal resection versus infliximab treatment of terminal ileitis in Crohn's disease: the LIR!C Trial. Gut. DOI: 10.1136/gutjnl-2018-317539. Feb 1, 2019.
20. McGain F, Muret J, Lawson C, Sherman JD. (2020) Environmental sustainability in anaesthesia and critical care. Br J Anaesth. DOI: 10.1016/j.bja.2020.06.055. Aug 12,2020.
21. Touw H, Stobernack T, Hunfeld NGM, Pickkers P. Size does matter. Sustainable choice of intravenous bags. Intensive Care Med. 2023;49(12):1529-1530. doi:10.1007/s00134-023-07240-3
22. Stilma W, Esmeijer A, Paulus F, Frenzel T, Touw H, Stobernack T. Open Versus Closed Suctioning in Invasively Ventilated Critically Ill Patients for Sustainability of ICU Care: A Life-Cycle Assessment Comparison. Respir Care. 2024;69(2):218-221. Published 2024 Jan 24. doi:10.4187/respcare.11189
23. Hellweg S, Milà i Canals L. (2014) Emerging approaches, challenges and opportunities in life cycle assessment. Science. DOI: 10.1126/science.1248361.
24. Life Cycle Assessment Best Practices of ISO 14040 Series Ministry of Commerce, Industry and Energy Republic of Korea Asia-Pacific Economic Cooperation Committee on Trade and Investment. 2004;
25. Goedkoop M, Heijungs R, Huijbregts MAJ, De Schryver A, Struijs J, and van Zelm R. 2009. ReCiPe 2008: A life cycle impact assessment method which comprises harmonised category indicators at the midpoint and endpoint levels. First edition. Report i: Characterization. the Netherlands: Ruimte en Milieu, Ministerie van Volkshuisvesting, Ruimtelijke Ordening en Milieubeheer.
26. Huijbregts MAJ, Steinmann ZJN, Elshout PMF, Stam G, Verones F, Vieira M, Zijp M, Hollander A, van Zelm R (2016) ReCiPe2016. A harmonized life cycle impact assessment method at midpoint and endpoint level. Report I: Characterization. RIVM Report 2016-0104. National Institute for Human Health and the Environment, Bilthoven.
27. Kim YE, Jung YS, Ock M, Yoon SJ. (2022) DALY Estimation Approaches: Understanding and Using the Incidence-based Approach and the Prevalence-based Approach. J Prev Med Public Health. DOI: 10.3961/jpmph.21.597.
28. Lee BK , Ellenbecker ML, Moure-Eraso R, Analyses of the recycling potential of medical plastic wastes. Waste Management, Volume 22, Issue 5, 2002, Pages 461-470, ISSN 0956-053X, https://doi.org/10.1016/S0956-053X(02)00006-5.
29. Conrardy J, Hillanbrand M, Myers S, Nussbaum GF. (2010) Reducing Medical Waste, AORN Journal, Volume 91, Issue 6, 2010, Pages 711-721, ISSN 0001-2092, https://doi.org/10.1016/j.aorn.2009.12.029.
30. Sharma R, Sharma M, Sharma R, Sharma V. (2013) The impact of incinerators on human health and environment. Reviews on Environmental Health. https://doi.org/10.1515/reveh-2012-0035
31. Asfaw S, Dilger A, Tummala N, Yates E. (2021). The intersection of climate change and surgery. Bull Am Coll of Surg.
32. Caycedo-Marulanda, A., Caswell, J. & Mathur, S. Comparing the environmental impact of anesthetic gases during transanal total mesorectal excision surgery at a tertiary healthcare centre. Can J Anesth/J Can Anesth **67**, 607–608 (2020). https://doi.org/10.1007/s12630-019-01527-0
33. Thiel CL, Eckelman M, Guido R, Huddleston M, Landis AE, Sherman J, Shrake SO, Copley-Woods N, Bilec MM. Environmental impacts of surgical procedures: life cycle assessment of hysterectomy in the United States. Environ Sci Technol. 2015 Feb 3;49(3):1779-86. doi: 10.1021/es504719g. Epub 2015 Jan 14. PMID: 25517602; PMCID: PMC4319686.
34. Yates EF, Bowder AN, Alexis N, Roa L, Velin L, Goodman AS, Nguyen LL, McClain CD, Meara JG, Cooper Z. (2021) Empowering Surgeons, Anesthesiologists, and Obstetricians to Incorporate Environmental Sustainability in the Operating Room. Ann. Surg. DOI: 10.1097/SLA.0000000000004755

**Tables and Figures legend**

Tables

- Table 1: Endpoint analysis

Figures

- Figure 1: System boundary
- Figure 2a: Tree map of the LCA of laparoscopic ICR for terminal ileitis in CD patients
- Figure 2b: Tree map of the LCA of laparoscopic STC in UC
- Figure 2c: Tree map of the LCA of LIFT procedures in Crohn’s PAF patients
- Figure 3a: Midpoint analysis of laparoscopic ICR for terminal ileitis in CD patients
- Figure 3b: Midpoint analysis of laparoscopic STC in UC patients
- Figure 3c: Midpoint analysis of LIFT procedures in Crohn’s PAF patients

**Supplementary Figures legend**

Supplementary Figures

- Supplementary Figure 1a: Process tree of the entire LCA of laparoscopic ICR for terminal ileitis in CD patients
- Supplementary Figure 1b: Process tree of the entire LCA of laparoscopic STC in UC patients
- Supplementary Figure 1c: Process tree of the entire LCA of LIFT procedures in Crohn’s PAF patients
- Supplementary Figure 2a: Process tree of the entire LCA of laparoscopic ICR for terminal ileitis in CD patients (without transport of personnel and patients)
- Supplementary Figure 2b: Process tree of the entire LCA of laparoscopic STC in UC patients (without transport of personnel and patients)
- Supplementary Figure 2c: Process tree of the entire LCA of LIFT procedures in Crohn’s PAF patients (without transport of personnel and patients)
- Supplementary Figure 3a: Endpoint analysis of laparoscopic ICR for terminal ileitis in CD patients – damage assessment
- Supplementary Figure 3b: Endpoint analysis of laparoscopic STC in UC – damage assessment
- Supplementary Figure 3c: Endpoint analysis of LIFT procedures in Crohn’s PAF patients – damage assessment
